# F‐group bZIPs in barley—a role in Zn deficiency

**DOI:** 10.1111/pce.13045

**Published:** 2017-09-19

**Authors:** Ahmad Zulhilmi Nazri, Jonathan H.C. Griffin, Kerry A. Peaston, Douglas G.A. Alexander‐Webber, Lorraine E. Williams

**Affiliations:** ^1^ Biological Sciences University of Southampton Southampton SO17 1BJ UK

**Keywords:** barley (*Hordeum vulgare*), bZIP transcription factors, zinc-deficiency response element (ZDRE), Zn deficiency, ZRT/IRT‐like protein (ZIP)

## Abstract

Zinc (Zn) deficiency negatively impacts the development and health of plants and affects crop yield. When experiencing low Zn, plants undergo an adaptive response to maintain Zn homeostasis. We provide further evidence for the role of F‐group transcription factors, AtbZIP19 and AtbZIP23, in responding to Zn deficiency in Arabidopsis and demonstrate the sensitivity and specificity of this response. Despite their economic importance, the role of F‐group bZIPs in cereal crops is largely unknown. Here, we provide new insights by functionally characterizing these in barley (Hordeum vulgare), demonstrating an expanded number of F‐group bZIPs (seven) compared to Arabidopsis. The F‐group barley bZIPs, HvbZIP56 and HvbZIP62, partially rescue the Zn‐dependent growth phenotype and ZIP‐transporter gene regulation of an Arabidopsis bzip19‐4 bzip23‐2 mutant. This supports a conserved mechanism of action in adapting to Zn deficiency. HvbZIP56 localizes to the cytoplasm and nucleus when expressed in Arabidopsis and tobacco. Promoter analysis demonstrates that the barley ZIP transporters that are upregulated under Zn deficiency contain cis Zn‐deficiency response elements (ZDREs). ZDREs are also found in particular barley bZIP promoters. This study represents a significant step forward in understanding the mechanisms controlling Zn responses in cereal crops, and will aid in developing strategies for crop improvement.

## Abstract 2

The heavy metal micronutrient Zinc (Zn) is crucial for all organisms, and therefore, crops grown in soils with low Zn availability yield less and have a reduced nutritional quality. Developing cereals that can maintain growth and yield on low Zn soils without the input of costly fertilizers, and accumulate more Zn in the edible parts, would have clear benefits for sustainable agriculture. F‐group bZIP transcription factors, AtbZIP19 and AtbZIP23, regulate the Zn‐deficiency response in Arabidopsis by upregulating a range of genes involved in Zn uptake and partitioning. Our molecular and genetic results suggest that the mechanisms that allow barley, an economically important cereal, to adapt to low Zn conditions are conserved. We show that there is an expanded number of F‐group bZIPs in barley (seven) and, from functional complementation and gene expression studies, provide evidence supporting a role for *HvbZIP56* and *HvbZIP62* in adapting to low Zn conditions.

## 1 INTRODUCTION

Zn is an essential mineral element for all life forms, required for growth and development, gene expression, and replication. This heavy metal micronutrient plays a significant role in metabolism of proteins, carbohydrates, lipids, and nucleic acids (Broadley, Brown, Cakmak, Rengel, & Zhao, 2012). Around 10% of the human proteome comprises Zn‐binding proteins (Andreini, Banci, Bertini, & Rosato, 2006; Clemens, Deinlein, Ahmadi, Horeth, & Uraguchi, 2013), and, therefore, even mild Zn deficiency is a key factor in malnutrition (Caulfield, de Onis, Blossner, & Black, 2004). Cereals serve as the main staple food for a large proportion of the world's population but have the shortcoming from a nutrition perspective of having low essential trace elements such as Zn in their edible tissues (Mikkelsen et al., 2012). This is a major issue for global human nutrition. In the past, the focus has been on strategies to increase crop yield; however, as micronutrient malnutrition affects around a third to a half of the world's population, especially women and pre‐school children (Gibson, 2006), we now recognize that enhancing the micronutrient content of edible plant foodstuffs is an urgent objective. To develop plant biotechnology strategies to improve Zn nutrition, we must understand the mechanisms underlying the efficiency of Zn uptake into the plant, distribution to shoot tissue, and partitioning of Zn to the grain. Furthermore, crops yield less and have a lower nutritional quality when grown in soils where Zn availability is low, further jeopardizing global food security (Genc et al., 2009; Ramesh et al., 2004). Deficiency of Zn on cultivated soils is a worldwide problem so developing crops that can maintain growth and yield under low soil Zn (Zn‐efficiency) without the input of costly fertilizers would have clear benefits for sustainable agriculture. Progress is being made in breeding programmes with the implementation of molecular markers, whereas genetic engineering has great potential to increase yield and nutritional quality under stress conditions (Sadeghzadeh, Rengel, & Li, 2015).

Zinc efficiency is complex, and the underlying molecular processes are not well understood in cereals. However, in the dicot model plant, Arabidopsis, the mechanisms employed in adapting to Zn‐deficient conditions have become clearer in recent years (Assunção et al., 2010; Inaba et al., 2015). Assunção et al. (2013) proposed that specific bZIP transcription factors (AtbZIP19 and AtbZIP23) respond to Zn deficiency by an, as yet, undefined mechanism. They suggested that it may involve release of Zn binding to cysteine/histidine (Cys/His)‐rich domains in these proteins under Zn‐deficient conditions. bZIP transcription factors would then regulate the transcription of Zn‐transporting ZIPs (Zrt/Irt‐like proteins) via binding to Zn deficiency response elements (ZDRE) in the promoters of these transporter genes. Upregulation of the ZIPs is proposed to increase Zn cellular levels in the cell, with some acting at the plasma membrane, functioning in Zn uptake (Assunção et al., 2010; Milner, Seamon, Craft, & Kochian, 2013). This is an adaptive mechanism to maintain Zn homeostasis.

bZIP proteins are one of the largest families of transcription factors, widely distributed in all eukaryotes. Characteristically, they contain a leucine zipper‐dimerization motif and a highly conserved basic domain, which are capable of binding DNA in a sequence‐specific manner. bZIPs bind to short palindromic or pseudopalindromic targets and can form homo‐ and/or hetero‐dimers. In plants, they regulate a diverse range of biological processes including light and hormone signalling, organ and tissue differentiation, plant senescence, and seed maturation (Choi, Hong, Ha, Kang, & Kim, 2000; Gao et al., 2014; Jakoby et al., 2002; Weisshaar, Armstrong, Block, Da Costa e Silva, & Hahlbrock, 1991). AtbZIP19 and AtbZIP23 are in the F group of bZIP transcription factors, a grouping based on phylogenetic analysis of a large number of angiosperm bZIP members dividing them into 13 groups (Jakoby et al., 2002; Correa et al., 2008). An Arabidopsis double mutant, *bzip19‐1 bzip23‐1*, showed a severe Zn‐deficiency phenotype when grown under low Zn conditions (Assunção et al., 2010). Transcriptomic analysis, comparing transcripts enhanced by Zn deficiency in wild‐type plants but not the double mutant, revealed a cohort of genes that showed differential regulation in response to Zn. Many of these genes contained one or more copies of the 10‐base pair imperfect palindrome ZDRE in their promoter region (Assunção et al., 2010). ZIP transporters and nicotianamine synthases were among the genes identified in this approach. In a proteomics analysis to identify AtbZIP19‐regulated proteins, ZIPs were again identified (AtZIP3 and AtZIP9) but also members of the defensin‐like family of proteins such as DEFL 203 (Inaba et al., 2015). Overall, these findings provided evidence that F‐group bZIPs, AtbZIP19 and AtbZIP23, coordinated the regulation of a cohort of genes to allow adaptation to Zn deficiency.

Clarifying the mechanisms involved in micronutrient sensing and signalling is important when developing strategies for sustainable agriculture. Barley is an economically important temperate cereal ranking fourth among cereals in farming acreage. As a diploid member of the grass family, barley is a natural model for the genetics and genomics of the Triticeae tribe (Schulte et al., 2009). ZIP transporter genes have been characterized in barley, and several are strongly induced by Zn deficiency (Tiong et al., 2015); however, we now need to decipher the molecular mechanisms for control. This study aims to provide insight into the role of F‐group bZIPs in this cereal crop in the adaptation to Zn deficiency and determine whether there is a conserved mechanism in monocots and dicots. We use bioinformatic analysis to identify and provide information for cloning the F‐group bZIPs from barley (Golden Promise). We isolated new barley bZIP transcription factors and tested whether they may be involved in adapting to Zn deficiency. Functional complementation in Arabidopsis was used to establish whether the mechanisms for responding to Zn deficiency may be conserved in monocots. Furthermore, F‐group bZIP transcriptional regulation in response to Zn deficiency was investigated, together with cellular localization. Regulatory sequences in barley ZIP transporters were analysed to determine whether ZDREs were present, as observed for the Arabidopsis Zn‐regulated ZIPs. Overall, we aimed to provide a more comprehensive understanding of the adaptation to Zn deficiency in this monocot crop.

## 2 MATERIALS AND METHODS

### 2.1 Plant growth

Soil grown Arabidopsis thaliana Columbia‐8, wild type and mutant seed were grown as previously described (Jaffé et al., 2012), with a day night cycle (23 °C, 16 hr light, 120 μmol m^−2^ s^−1^; 18 °C, 8 hr dark). To assess the metal sensitivity of Arabidopsis mutant/transgenic lines, agarose plate assays were conducted as previously described (Menguer et al., 2013; Mills et al., 2008) using one‐half‐strength Murashige and Skoog medium (0.5 MS; Murashige & Skoog, 1962). Various metals (e.g. ZnSO_4_, CuSO_4_, FeNaEDTA or MnSO_4_) were included or omitted to test their effects. Seeds were stratified in the dark at 4 °C for 48 hr prior to transfer to a controlled‐environment cabinet (22 °C, 16 hr light, 120 μmol m^−2^ s^−1^; 18 °C, 8 hr dark) and plates were incubated vertically. To grow Arabidopsis hydroponically, seeds were placed in 1.5 ml Eppendorf tubes with lids and bottom removed, containing 0.5% agarose (Melford, UK). They were placed in polyethylene foam floats, positioned on top of the nutrient solution in hydroponic tubs. Seed were stratified as above prior to transfer to a controlled‐environment room under short day conditions (23 °C, 8 hr light 120 μmol m^−2^ s^−1^; 18 °C, 16 hr dark). The nutrient solution was based on Maathuis et al. (2003) containing the following composition for control conditions: 1.25 mm KNO_3_, 500 μm Ca(NO_3_)_2_.4H_2_O, 0.5 mm MgSO_4_.7H_2_O, 42.5 μm FeNaEDTA, 0.625 mm KH_2_PO_4_, 2 mm NaCl, 0.16 μm CuSO_4_.5H_2_O, 0.38 μm ZnSO_4_.7H_2_O, 1.8 μm MnSO_4_.H_2_O, 45 μm H_3_BO_3_, 0.015 μm (NH_4_)_6_Mo_7_O_24_.4H_2_O, and 0.01 μm CoCl_2_.6H_2_O). Zn was excluded from the media for low Zn conditions. The media were changed once after 2 weeks and once every week after that. The plants were harvested after 40 days in the growth chamber.

To grow hydroponically, *Hordeum vulgaris* L. cv Golden Promise seed was sterilized in 1% bleach for 15 min, rinsed in sterile water, germinated on wet tissue paper for 5 days, and then individual seedlings were grown in aerated 1 l hydroponic culture pots (Thermo Fischer Scientific, UK) in a controlled‐environment room (21 °C, 16 hr light, 220 μmol m^−2^ s^−1^, 55% humidity; 16 °C, 8 hr dark, 65% humidity). Pots were filled with a nutrient solution as described in Lombnaes and Singh (2003), which contained 2 mm Ca(NO_3_)_2_, 1 mm KNO_3_, 80 μm KH_2_PO_4_, 0.5 mm MgSO_4_, 0.01 mm H_3_BO_3_, 0.9 mm NaOH, 75 μm Fe(NO_3_)_3_, 8.0 μm ZnCl_2_, 0.6 μm MnCl_2_, 2.0 μm CuCl_2_, 0.1 μm NiCl_2_, 0.1 μm Na_2_MoO_4_, and 1 mm HEDTA buffered at pH 6.0 with 1.0 mm 2‐[N‐morpholino]ethanesulfonic acid. For the Zn‐deficient treatment, Zn was omitted from the media. The nutrient solution was replaced every 3 days, and, at day 14, KH_2_PO_4_ was increased to 160 μm, as described previously (Lombnaes & Singh, 2003). Root and shoot were harvested separately either for fresh weight determinations or for freezing in Liquid N_2_ for subsequent RNA extraction.

### 2.2 Genomic DNA and RNA isolation and cDNA synthesis

Genomic DNA was isolated from Arabidopsis and barley using the DNAmite plant genomic DNA extraction kit (Microzone, UK) according to manufacturer's instructions. Total RNA was extracted from Arabidopsis using a phenol‐SDS extraction and LiCl precipitation method based on Verwoerd, Dekker, and Hoekema (1989) or by using Trizol Reagent according to manufacturer's instructions (Invitrogen Life Technologies RNA, UK). RNA from hydroponically grown barley was also isolated using the Trizol method. First‐strand cDNA synthesis using 1 μg of total RNA was carried out using the ImProm‐II™ reverse transcriptase kit (Promega, USA) with an oligo(dT) primer according to manufacturer's instructions.

### 2.3 PCR and real‐time PCR

Standard 10 μl PCR reactions were performed using BioMix Red (Bioline), forward (F) and reverse (R) primers (Table S1) with either genomic DNA or cDNA. Reactions were performed in a peqSTAR 96 Universal PCR machine (Peqlab, Germany). Real‐time PCR was performed as described previously (Jaffé et al., 2012) with specific forward and reverse primers (Table S1). For Arabidopsis, multiple plants were pooled from a particular genotype to generate 1 μg/μl of RNA for each biological replicate, which was then used to generate cDNA for gene expression comparisons. For barley, tissue from three plants were pooled for each biological replicate. Gene expression levels were calculated on the basis of Pfaffl (2001), standardized by normalizing to *HvRNABP* for barley (Mikkelsen et al., 2012) or *AtSAND* for Arabidopsis (Remans et al., 2008), and analysed using Opticon software. They were expressed relative to levels at day 0 of the treatment in barley or to expression in Zn‐sufficient wild type in Arabidopsis, which were both expressed as 1. The primers used for monitoring expression of a range of genes are given in Table S1.

### 2.4 Isolation of insertional mutants for AtbZIP19 and AtbZIP23

Mutant isolation was as described previously (Mills et al., 2005; Mills et al., 2008). Seed for putative T‐DNA insertion lines for *AtbZIP19* and *AtbZIP23* from the Salk collection (http://signal.salk.edu) were obtained from the Nottingham Arabidopsis Stock Centre. The seed lines were N667534, N506692, and N657869 (*bzip19‐1*, *19‐2*, and *19‐4*, respectively), and N656437 and N653060 (*bzip23‐1* and *23‐2*, respectively). To isolate plants homozygous for the insert, the plants were genotyped with respect to the T‐DNA insert using PCR on genomic DNA with two primers for the T‐DNA left border (LBa1 and LBb1) and gene‐specific primers: bZIP19R2 for all *bzip19* mutants and bZIP23R2 for all *bzip23* mutants (Table S1). PCR was performed at 94 °C for 2 min followed by 38 cycles of 94 °C for 30 s, 58 °C for 1 min, 72 °C for 1 min, then a final elongation step of 72 °C for 5 min. The absence of wild‐type products was confirmed using bZIP19F2 and bZIP19R2 for *AtbZIP19*, and bZIP23F2 and bZIP23R2 for *AtbZIP23* with the same conditions above. The insert position was confirmed by sequencing the product obtained with the T‐DNA border and gene‐specific primer. The lack of a transcript was confirmed at the RNA level by RT‐PCR using primers bZIP19F2 and bZIP23R2 for *AtbZIP19* and bZIP23F2 and bZIP23R2 for *AtbZIP23*. Amplification of *Actin2* was used as a positive control using primers Actin2.F and Actin2.R with the PCR conditions as above. Double mutants were obtained by crossing the various singles and selecting for plants homozygous for both mutant alleles using PCR as above. These plants were confirmed to be double mutants at the RNA level by RT‐PCR, as above.

### 2.5 Bioinformatic analysis to identify barley F‐group bZIP sequences

The OsbZIP48/53 amino acid sequence was retrieved from the plant transcription factor database V2.0 (PlantTFDB) (http://planttfdb_v2.cbi.edu.cn/). To find barley genomic sequence related to the rice bZIP, a blast search was done using the assembly_WGSBarke/Bowman/Morex databases (Mayer et al., 2012). The OsbZIP48/53 coding sequence was aligned with the resulting contigs database to predict an open reading frame using Clustal Omega (http://www.ebi.ac.uk/Tools/msa/clustalo/). For every contig analysed, the resulting predicted barley bZIP was BLASTed against the database above to find any additional related *HvbZIPs*. Additionally, all predicted genes were BLASTed against full‐length cDNA database (Matsumoto et al., 2011), and HC_genes_CDS_Seq and LC_genes_CDS_Seq databases (Mayer et al., 2012). All databases mentioned were accessed in the IPK BLAST server (http://webblast.ipk‐gatersleben.de/barley/viroblast.php ​), part of The International Barley Sequencing Consortium (IBSC).

### 2.6 Phylogenetic analysis of bZIPs

The evolutionary history was inferred using the neighbour‐joining method (Saitou & Nei, 1987) as previously described (Jaffé et al., 2012). The bootstrap consensus tree, inferred from 1,000 replicates, is taken to represent the evolutionary history of the taxa analysed and was generated using MEGA7 (http://www.megasoftware.net/) (Kumar, Stecher, & Tamura, 2016). The multiple sequence alignment was made with ClustalW module within MEGA7 using default parameters.

### 2.7 Cloning barley *bZIPs*

The seven F‐group *HvbZIPs* identified from bioinformatics analysis were cloned using gateway technology. First, *HvbZIPs* were amplified from barley cDNA (Golden promise) with the relevant primers (Table S1) using PCR with Pfu polymerase (Promega, UK). They were cloned into pENTR/D‐TOPO vector (Invitrogen, UK) and then transferred to pMDC32, and in the case of *bZIP56* also into pMCD83 (Curtis & Grossniklaus, 2003) by LR recombination. These vectors allow constitutive expression under the 35S promoter and pMDC83 provides a GFP tag at the C‐terminus. Expression clones were sequenced to confirm.

### 2.8 Expressing barley bZIPs in Arabidopsis

Barley *HvbZIP56* and *HvbZIP62* in pMCD32 and *HvbZIP56* in pMDC83 were transformed into *Agrobacterium tumefaciens* GV3101 by electroporation. *bzip19 bzip23* double mutants or wild‐type plants were stably transformed using the floral dip method (Clough & Bent, 1998) as previously described (Mills et al., 2010). Segregation analyses at the T2 and T3 stages were performed to isolate single‐insertional homozygous transgenic plants. Homozygous T3 plants were used for analysis. Multiple independent lines were generated and at least two independent lines for each construct were examined in detail. RT‐PCR was used to confirm expression of the appropriate construct in the different lines using primers specific for the particular bZIP (Table S1).

### 2.9 Elemental analysis

Elemental analysis was determined using inductively coupled plasma atomic emission spectroscopy on Arabidopsis lines grown as described above in the metal‐sensitivity plate assays. Briefly, plants were grown on 0.5 MS with 0 or 15 μm Zn for 21 days, harvested and dried to constant dry weight and then subjected to acid digestion. At least 50 mg dry weight of tissue was used for each replicate derived from seedlings pooled from a number of plates.

### 2.10 Subcellular localization of HvbZIP56

*HvbZIP56* cloned in *pMDC83,* which tags GFP at the C‐terminus (Curtis & Grossniklaus, 2003), was expressed in *bzip19‐4 bzip23‐2* as above. Transgenic plants (T3 generation) were used to investigate subcellular localization and two independent transgenic lines were tested. At least three replicate seedlings were analysed for each condition and representative images are shown. Transient expression in tobacco (Nicotiana tabacum, ‘Petit Havana’) was carried out as previously described (Brandizzi, Snapp, Roberts, Lippincott‐Schwartz, & Hawes, 2002; Siemianowski et al., 2013). Several different plants were used for transient expression and at least two leaves were transfected for each. LTI6b‐mOrange2 in pB7FWG2 was used as a marker for the plasma membrane and Hoechst 3342 (Thermo Fischer Scientific) at 10 mg ml^−1^ was used for nuclear staining. Plants were analysed by confocal laser scanning microscopy (Leica TCS SP8 Confocal) with GFP excitation generally at 488 nm and detection at 500–550 nm. LTI6b‐mOrange2 was detected using 561 nm excitation and 563‐590 nm emission and Hoechst at 405 nm excitation and 450 nm emission.

### 2.11 Statistical analysis

Two‐way ANOVA was used for statistical analysis, conducted using Minitab and Prism software, with the threshold for statistical significance difference taken at a 95% confidence interval. Tukey's HSD or Fisher's Least Significant Difference (LSD) post hoc tests were used as indicated to determine significant differences; values with the same letter on graphs are not significantly different (*P*≤ 0.05).

## 3 RESULTS

### 3.1 Identifying and cloning F‐group bZIPs from barley

**Figure 1:**
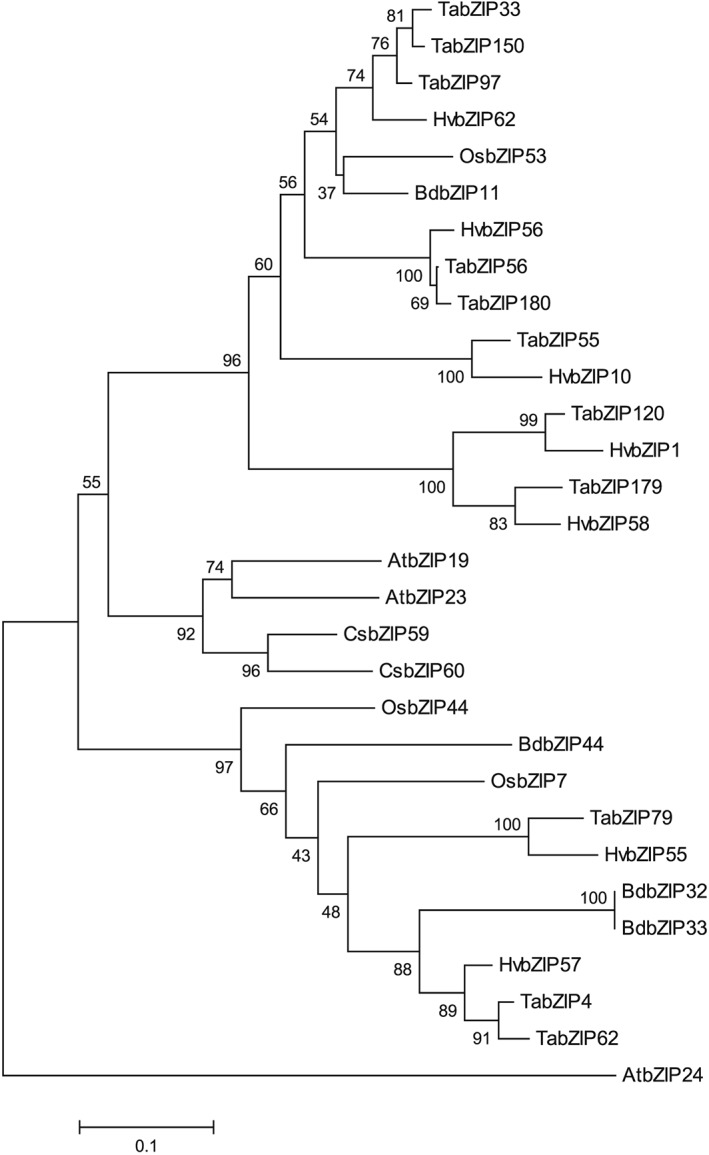
Phylogenetic analysis of F‐group bZIP proteins from Hordeum vulgare and a range of other species. The nonrooted, bootstrapped plot was constructed using MEGA 7 (Kumar et al., 2016) with a multiple alignment of bZIPs from H. vulgare, Triticum aestivum, Brachypodium distachyon, Oryza sativa, Arabidopsis thaliana, and Cucumis sativus. The percentage of replicate trees in which the associated taxa clustered together in the bootstrap test (1,000 replicates) are shown next to the branches (Felsenstein, 1985). The tree is drawn to scale, with branch lengths in the same units as those of the evolutionary distances used to infer the phylogenetic tree. The evolutionary distances were computed using the Poisson correction method (Zuckerkandl & Pauling, 1965) and are in the units of the number of amino acid substitutions per site. All positions containing gaps and missing data were eliminated. Accession numbers and identifier of the predicted proteins are listed in Table S3

The rice sequence *OsbZIP48/53,* which is closely related to Arabidopsis F‐group bZIPs, *bZIP19* and *bZIP23* (Assunção et al., 2010), was used to search barley contigs to identify prospective F‐group barley bZIP orthologues (Table S2). From this and subsequent searches with the resulting barley bZIPs, we identified seven F‐group bZIPs (*HvbZIP1*, *10, 55, 56, 57, 58*, and *62*; Table S2). These seven barley F‐group sequences are shown in an unrooted phylogenetic tree (Figure 1, Table S3), together with F‐group bZIP protein sequences previously identified from rice, Brachypodium, wheat, Arabidopsis and cucumber (Assunção et al., 2010; Baloglu, Eldem, Hajyzadeh, & Unver, 2014; Li et al., 2015; Liu & Chu, 2015; Pourabed, Golmohamadi, Monfared, Razavi, & Shobbar, 2015). The presence of both monocot and dicot representatives in this clade of bZIPs indicates a conserved evolution and could imply that there is conservation of function. The wheat F‐group bZIPs are the most closely related sequences to the barley bZIPs and for some of the wheat F group, several homeologs are included, according to Li et al. (2015). Ninety‐six Brachypodium bZIP genes have been identified and three (BdbZIP11, BdbZIP32, and BdbZIP33) were shown to group with Arabidopsis F‐group bZIPs in phylogenetic analysis (Liu & Chu, 2015). BdbZIP11 is referred to as BdbZIP10 by Glover‐Cutter, Alderman, Dombrowski, and Martin (2014) and this has been implicated in oxidative stress and Zn‐deficiency responses in Brachypodium. HvbZIP62 and HvbZIP56 are most closely related to BdbZIP11 (76% and 65% identity respectively; Table S4). Using our sequence predictions from information available from Morex and Bowman cultivars (Table S2), we amplified and cloned these seven F‐group bZIPs from cDNA prepared from the barley cultivar Golden Promise, demonstrating they are expressed. Each of the Golden Promise bZIP sequences were identical or highly similar to corresponding sequences found or predicted in the other barley cultivars (Table S2). An additional sequence, AK361769.1 (mRNA)/BA92973 (protein), was previously reported as an F‐group bZIP and named HvbZIP61 (Pourabed et al., 2015). However, we did not find it in an analysis of barley contigs and no orthologue was found in the wheat or rice genomes. Furthermore, sequence comparisons and phylogenetic analysis show that it is a more divergent sequence having less than 11% identity to the other F‐group bZIPs (Figure S1 and Table S4). The results from BLAST showed that it was 95% identical to a sequence XP_008187462 from Acyrthosiphon pisum (pea aphid), suggesting that when the original libraries were prepared in the study of Matsumoto et al. (2011) this sequence may have been aphid in origin.

The seven F‐group barley bZIPs range in predicted molecular weight from 19.9 to 27.5 KDa (see Table S2) and vary in their percentage identity (Table S4). HvbZIP56 and HvbZIP62 are closely related (65% identity), and they show the highest identity to AtbZIP19 and AtbZIP23. When aligned together with Arabidopsis F‐group bZIPs, it can be seen that all seven barley F‐group bZIPs possess the bZIP domain and all except HvbZIP10 contain the Cys/His rich domains 1 and 2 (Figure S2).

### 3.2 Additional unique Arabidopsis bzip19 bzip23 double mutants are adversely affected by Zn deficiency

**Figure 2:**
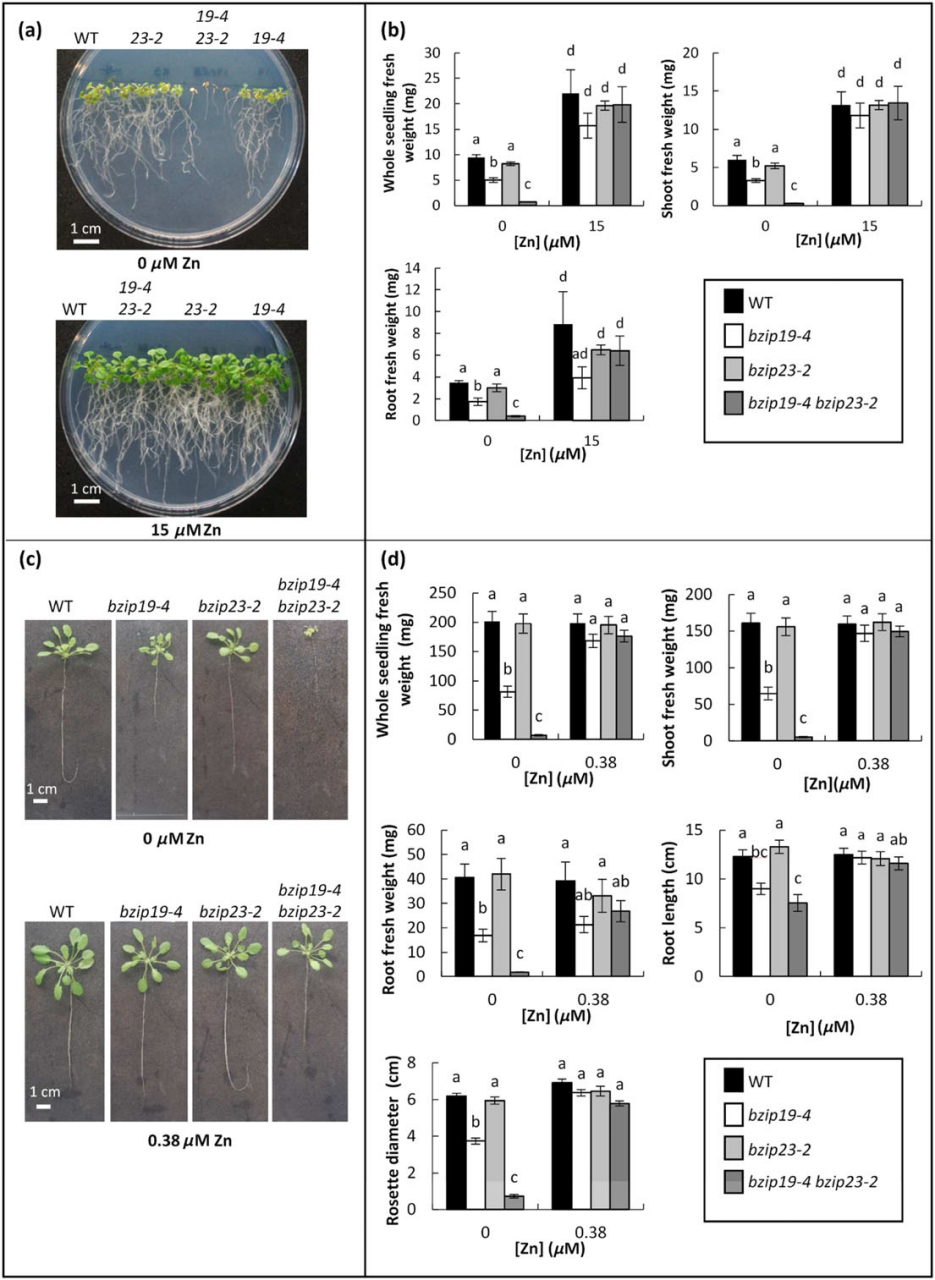
Zn‐deficiency phenotype of the *bzip19‐4 bzip23‐2* double mutants in plate and hydroponic assays. Wild type (WT), *bzip19‐4*, *bzip23‐2*, and *bzip19‐4 bzip23‐2* T‐DNA insertion Arabidopsis thaliana mutants grown on agarose 0.5 MS plates for 21 days with 0 μm Zn or 15 μm Zn (a,b) or hydroponic culture for 40 days with 0 μm Zn or 0.38 μm Zn (c,d). For each growth condition, fresh weight measurements for whole seedling, shoot and root are shown. Root length and rosette diameter are also shown for the hydroponically grown plants. For the plate assays, the data are on the basis of the means from six plates (±SEM) with four seedlings per line, per plate, each plate containing four plant lines. For hydroponic assays, the data were based on 30 plants (±SEM). Means not sharing a letter are significantly different (*P* ≤ 0.05); Tukey's post hoc test

To test whether there is conservation of function between Arabidopsis (dicot) and barley (monocot) F‐group bZIPs, a functional complementation approach was taken. For this we generated several new Arabidopsis *bzip19 bzip23* double mutants. There has been some conflicting information in the nomenclature of the single mutants isolated in previous studies (Assunção et al., 2010; Inaba et al., 2015). For clarification we show in Table S5 the naming according to these two studies and the nomenclature adopted in this study, which adheres to that in the original study by Assunção et al. (2010). We have independently isolated Arabidopsis *bzip19‐1*, *bzip19‐2* and *bzip19‐4* and also *bzip23‐1* and *bzip23‐2*. More precise information on the position of the inserts is given in Figure S3a and b. We also generated four unique double mutant alleles (*bzip 19‐4 bzip 23‐2*, *bzip19‐2 bzip23‐1*, *bzip19‐4 bzip23‐1*, and *bzip19‐1 bzip23‐2*) as well as independently generating *bzip19‐1 bzip23‐1* for comparison (Figure S3a and b). The novel double mutants lacked full‐length transcripts for both *AtbZIP19* and *AtbZIP23,* and were thus considered knockout mutants (Figure S3a,b). All these double mutant combinations showed a clear Zn‐deficiency phenotype (stunting and chlorosis) when grown on agarose plates and in hydroponics (Figure 2a,b; Figure S4). Plants grown at 15 μm Zn (control conditions) displayed no significant differences between the genotypes.

Previously when grown on agar plates for a similar time it was reported that only the *bzip19‐1 bzip23‐1* double mutant was strongly affected by low Zn and not the *bzip19‐1* and *bzip23‐1* single mutants (Assunção et al., 2010). The same mutant alleles (*bzip19‐1* and *bzip23‐1*) were independently isolated here and compared directly on plates under similar conditions with the *bzip19‐4, bzip23‐2* mutant alleles; they responded very similarly (Figure S5). The media used for this comparison is 0.5 MS whereas Assunção et al. (2010) used full MS. In summary, under our conditions on plates (0.5 MS) all *bzip19* mutant alleles (*bzip19‐1*, *19‐2*, *19‐4*) showed a stronger Zn‐deficiency phenotype compared to wild type while the single *bzip23‐1* and *23‐2* mutant alleles were not significantly different to wild type. In all mutant combinations, the double mutants were more strongly inhibited than wild type and *bzip19* single mutants.

**Figure 3:**
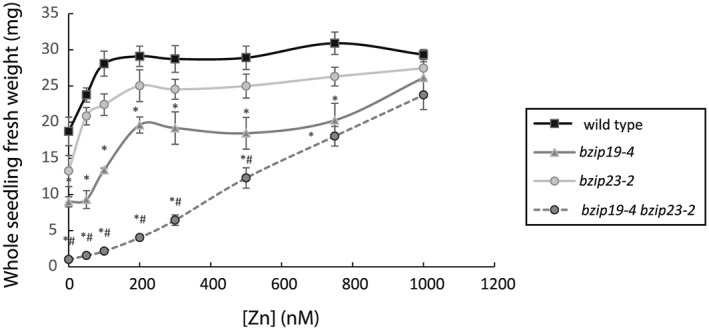
Zn‐concentration dependency of the *bzip19‐4 bzip23‐2* double mutant. Wild type, *bzip19‐4* and *bzip23‐2* single mutants and *bzip19‐4 bzip23‐2* double mutants were grown alongside on 0.5 MS media containing a range of Zn concentrations for 19 days. Total fresh weight is shown on the basis of means from six plates (±SEM) with four seedlings per line, per plate, each plate containing four plant lines. *, *P* ≤ 0.05 = significantly different to the mean of wild type. #, *P* ≤ 0.05 = significantly different to the mean of *bzip19‐4* and *bzip23‐2*.

It was important to determine the concentration of Zn that rescued the Arabidopsis *bzip* double mutants as this has not been investigated previously and it reveals information about the sensitivity of the response. As little as 1 μm Zn restored the growth of *bzip19‐4* single and *bzip19‐4 bzip23‐2* double mutant to wild‐type levels (Figure 3).

### 3.3 Other micronutrient deficiencies (Mn, Fe, and Cu) do not impact the response of the bzip double mutants compared to wild type.

**Figure 4:**
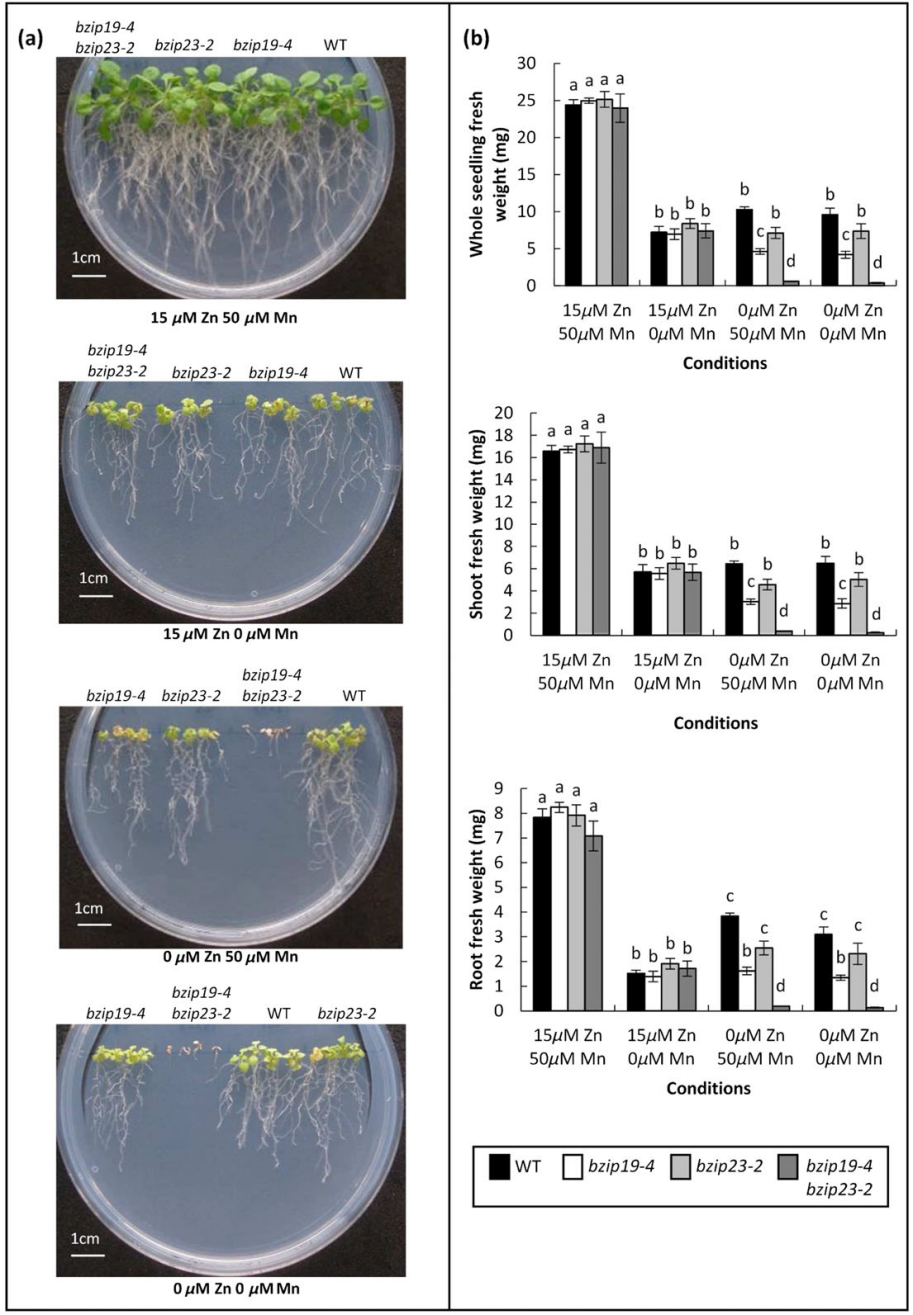
Mn deficiency does not influence the response of the *bzip19*, *bzip23*, and *bzip19 bzip23* mutants to Zn deficiency nor specifically affect these mutants compared to wild type. (a) Wild type (WT), *bzip19‐4*, *bzip23‐2*, and *bzip19‐4 bzip23*‐2 mutants grown on 0.5 MS media under a range of Zn and Mn concentrations for 21 days. (b) Whole seedling, shoot and root fresh weight is shown, the data are based on the means from six plates (±SEM) with four seedlings per line, per plate, each plate containing four plant lines. Means not sharing a letter are significantly different (*P* ≤ 0.05); Tukey's post hoc test

To test the specificity of the response of the Arabidopsis *bzip* double mutants, they were grown under Mn, Cu and Fe deficiency. Results for the response of the mutants to Mn deficiency are shown in Figure 4a,b. Wild type, single and double mutants were grown on 0 μm Zn media or 15 μm Zn media in combination with 0 μm Mn or 50 μm Mn. Fresh weight of seedlings was reduced at 15 μm Zn 0 μm Mn compared to control conditions (15 μm Zn 50 μm Mn) but in this case the *bzip* mutants responded similarly to wild type. In addition, there was no influence of Mn on the Zn‐deficiency response observed in these mutants as they showed similar responses in the presence or absence of Mn under 0 μm Zn conditions. Similarly, no significant effect was found in the response of the double mutants compared to wild type when grown under Cu or Fe deficiency unless they were also under Zn deficiency (Figure S6); thus the deficiency responses observed in the double mutants is specific to low Zn exposure.

### 3.4 Barley *HvbZIP56* and *HvbZIP62* partially restore the Zn‐deficiency phenotype of Arabidopsis *bzip19 bzip23* double mutants

**Figure 5:**
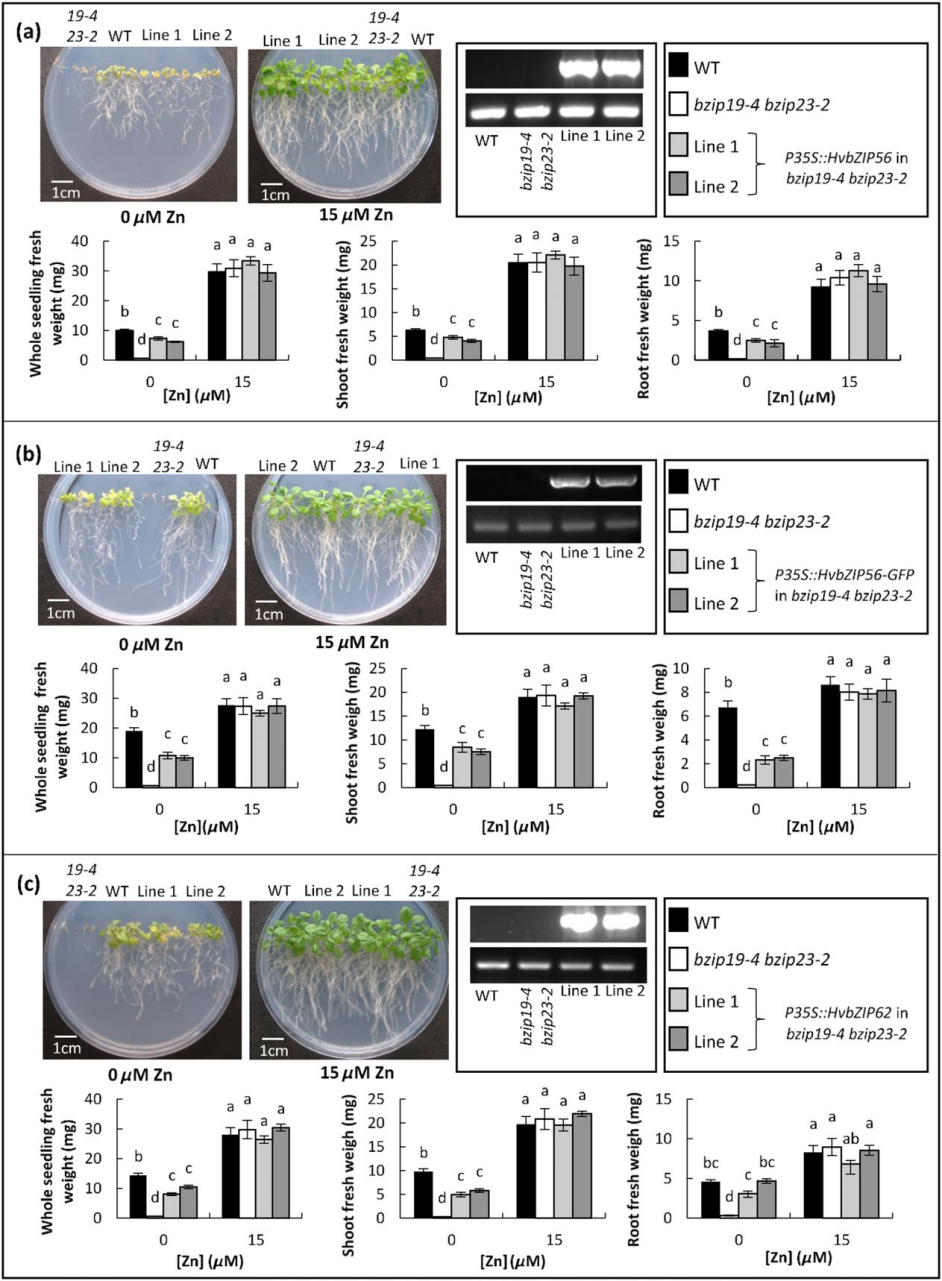
HvbZIP56, HvbZIP56‐GFP, and HvbZIP62 partially rescue the Zn‐deficiency phenotype of the Arabidopsis *bzip19‐4 bzip23‐2* double mutant. HvbZIP56, HvbZIP56‐GFP, and HvbZIP62 were expressed under the control of the 35S promoter in the *bzip19‐4 bzip23‐2* double mutant. RT‐PCR is shown for different lines with the upper panel showing expression in the respective lines of *HvbZIP56* (a), *HvbZIP56‐GFP* (b), and *HvbZIP62* (c) and the lower panel showing *ACTIN2* as a control; wild type and *bzip19‐4 bzip23‐2* lines were included as controls. The growth of two independent transgenic lines for each construct are shown together with wild type and *bzip19‐4 bzip23‐2* mutants on 0.5 MS media for 21 days with 0 μm Zn or 15 μm Zn. The whole seedling, shoot and root fresh weight data are based on the means from six plates for each condition (±SEM) with four seedlings per line, per plate, each plate containing four plant lines. Means not sharing a letter are significantly different (*P* ≤ 0.05); Tukey's post hoc test

**Figure 6:**
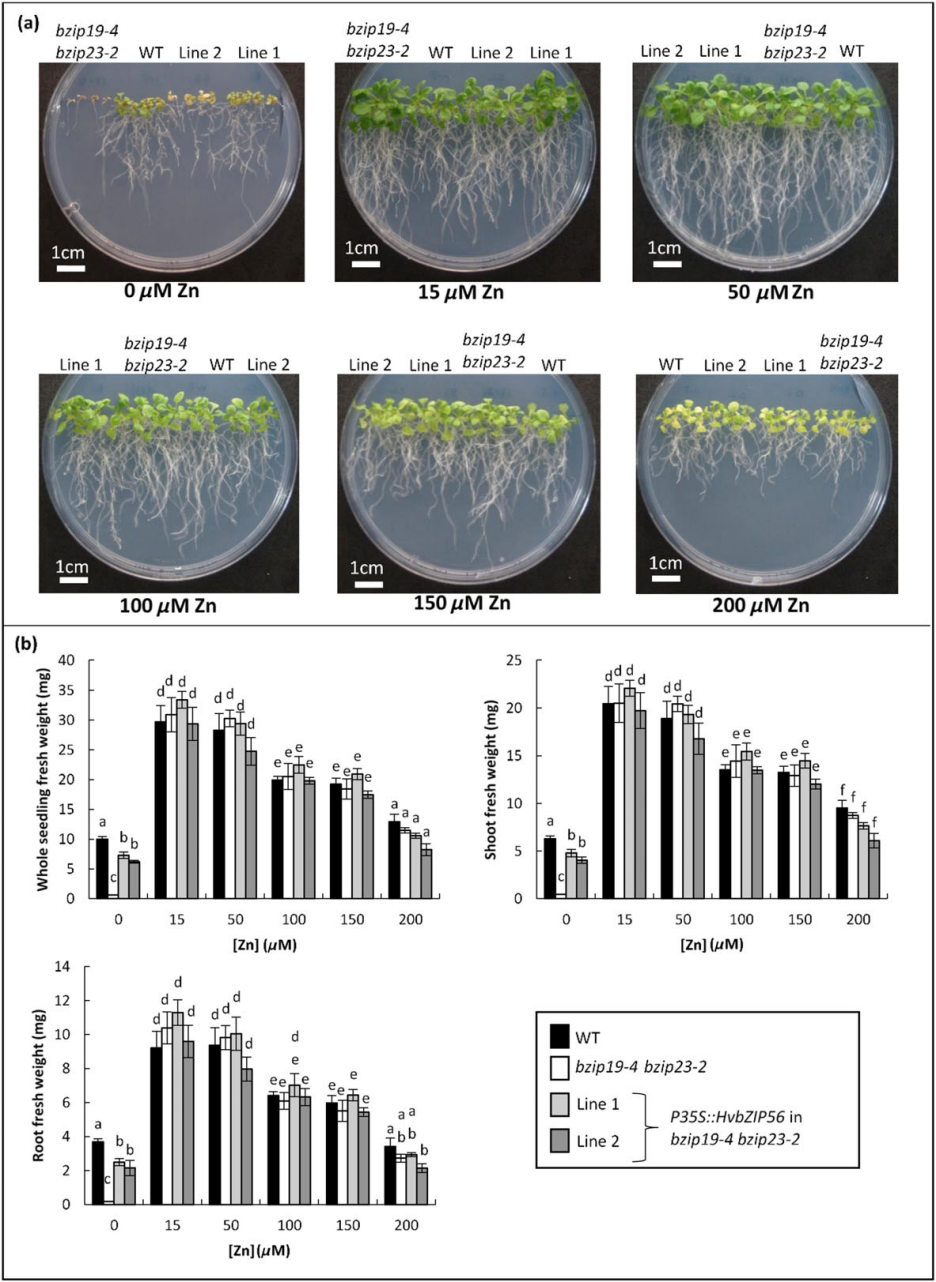
HvbZIP56 has little influence on the Arabidopsis *bzip19‐4 bzip23‐2* mutant at elevated Zn. Wild type (WT), *bzip19‐4 bzip23‐*2 mutants, and two independent transgenic double mutant lines expressing *HvbZIP56* were grown on 0.5 MS media for 21 days with a range of Zn concentrations. Whole seedling, shoot and root fresh weight are shown. The data are based on means from six plates (±SEM) with four seedlings per line, per plate, each plate containing four plant lines. Means not sharing a letter are significantly different (*P* ≤ 0.05); Tukey post hoc test

Taking advantage of the clear Zn‐deficiency growth phenotype in the Arabidopsis double mutants, they were used as a useful tool for evaluating the function of barley F‐group bZIPs and to confirm a potential role for the latter in the Zn‐deficiency‐response mechanism. To determine whether there is conservation of function between Arabidopsis and barley bZIPs, *HvbZIP56* and *HvbZIP62* expressed under the 35S promoter were tested for their ability to complement the Zn‐deficiency response of the *bzip19‐4 bzip23‐2* double mutant*. HvbZIP56*‐GFP was also tested in order to use it for further localization studies. Multiple lines were generated and two independent transgenic lines showing clear expression of the particular barley bZIP (Figure 5) were characterized in detail. Comparable levels of growth were observed in all lines at standard Zn (15 μm) and higher concentrations (Figures 5 and 6). When grown under Zn deficiency (0 μm Zn), *HvbZIP56*, *HvbZIP56‐GFP*, and *HvbZIP62* rescued the growth of the Arabidopsis double mutant, but not quite to wild‐type levels (Figure 5). The lack of complete rescue was not due to differing metal micronutrient levels as the complemented lines showed similar levels to wild type (Figure S7). All genotypes had a very low Zn content, which was unsurprising as there was no Zn added to the growth medium. Interestingly growth on the Zn‐deficient medium caused a significant reduction in the Fe content of the double mutant compared to wild type and this was restored in the *HvbZIP56*‐expressing lines (Figure S7).

As there was incomplete growth rescue, *HvbZIP56*‐expressing double mutant plants were compared to the *bzip19* single mutant and growth was restored to a similar level (Figure S8). Additionally *HvbZIP56*‐expressing wild‐type Arabidopsis lines were generated but no increase in resistance to Zn deficiency was found, nor any effect at elevated Zn compared to wild‐type controls (Figure S9).

### 3.5 HvbZIP56 induces expression of ZIP transporters when expressed in Arabidopsis bzip19 bzip23

**Figure 7:**
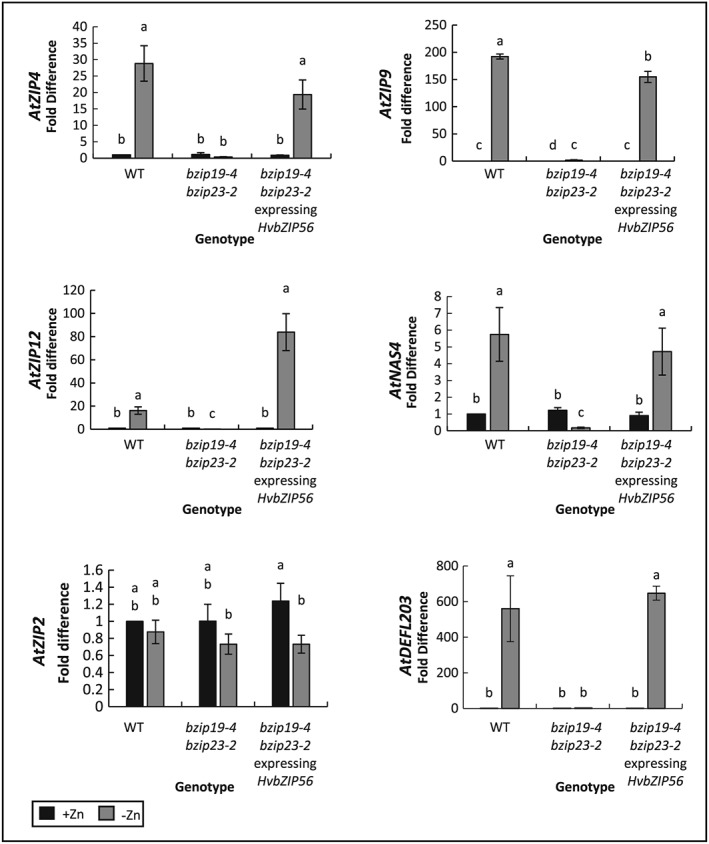
HvbZIP56 restores gene expression in response to Zn deficiency in the Arabidopsis *bzip19‐4 bzip23‐2* mutant. Real‐time PCR to compare Arabidopsis gene expression in response to Zn deficiency in wild type (WT), *bzip19‐4 bzip23‐2* double mutant, and *HvbZIP56‐*expressing *bzip19 bzip23* line 1. Seedlings were grown for 3 weeks on MS medium with Zn (black) or without Zn (grey). *SAND* was used for normalization. The expression levels were relative to that in wild‐type (WT) seedlings grown with Zn, which was expressed as 1. Data presented are means (±SEM) of three biological replicates. Means not sharing a letter are significantly different (*P* ≤ 0.05); Fisher's least significant difference post hoc test

To investigate the mechanism further, we tested whether the restoration of growth on low Zn seen in the complemented double mutant lines correlated with an up‐regulation of ZIP transporters. *At* *ZIP9* and *At* *ZIP12* were investigated initially as these have previously been demonstrated to be upregulated by AtbZIP19 and AtbZIP23 respectively under Zn deficiency (Inaba et al., 2015). *AtZIP2* was used as a control as previously this did not show marked up‐regulation under these conditions (Assunção et al., 2010). Expression of HvbZIP56 in the double mutant led to a strong up‐regulation of *At* *ZIP9* and *At* *ZIP12* expression but not *At* *ZIP2* under Zn deficiency (Figure 7). Other genes previously shown to be regulated by AtbZIP19 and 23 in Arabidopsis included the Zn transporter *At* *ZIP4,* and non‐transporter genes *At* *NAS4* and *At* *DEFL203* (Assunção et al., 2010; Inaba et al., 2015); these were also upregulated in the HvbZIP56‐expressing double mutant (Figure 7). Expression of HvbZIP62 in the double mutant also resulted in up‐regulation of *AtZIP9* and *AtNAS4* (Figure S10).

### 3.6 Regulation of bZIPs in barley

**Figure 8:**
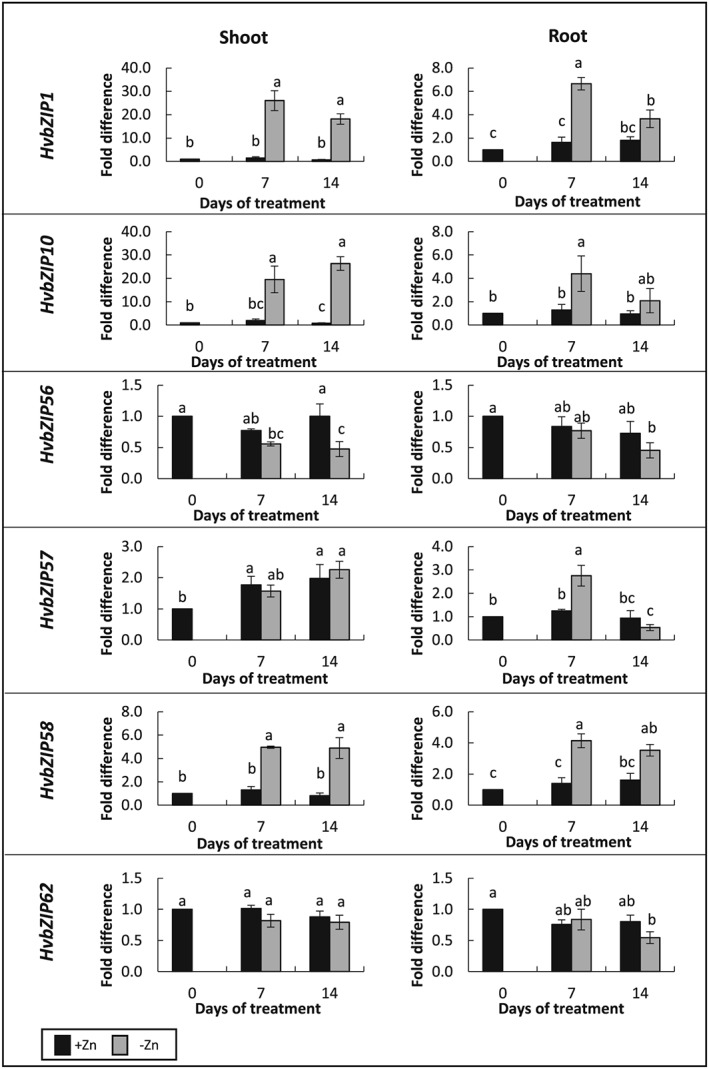
Regulation of bZIPs in barley following imposition of Zn‐deficiency conditions. Real‐time PCR to determine gene expression of barley *bZIPs* in roots and shoots of barley over time in response to Zn deficiency. *RNABP* was used for normalization. Data are means (±SEM) of three biological replicates. Black bar and grey bar indicate gene expression level under 8 μm Zn (+Zn) and 0 μm Zn (−Zn) conditions, respectively. The expression levels are relative to expression at day 0 of the treatment, which was expressed as 1. Means not sharing a letter are significantly different (*P* ≤ 0.05); Fisher's least significant difference post hoc test

A key question is whether F‐group bZIPs themselves are regulated by Zn deficiency. Initially, Assunção et al. (2010) reported around twofold up‐regulation of Arabidopsis *AtbZIP19* and *23* under Zn deficiency but later they indicated that they may not be significantly Zn regulated (Assunção et al., 2013). Therefore, it was important to determine whether cereal F‐group bZIPs are Zn regulated. We imposed the conditions of Lombnæs *&* Singh (2003) that were shown to induce Zn deficiency in barley. Symptoms of Zn deficiency started to become apparent at 7 days after treatment, in terms of reduction in fresh weight of shoots (Figure S11). By 14 days both shoot and root fresh weights were reduced and leaves showed chlorosis and withering at the tips. To establish that Zn‐deficiency was affecting gene expression of particular *HvZIPs*, we measured the response of *HvZIP5*, *HvZIP10*, *HvZIP13*, and *HvZIP14*. Up‐regulation of *HvZIP5, Hv*ZIP10, *HvZIP13* (but not *HvZIP14*) was observed under Zn deficiency (Figure S12) and these results are consistent with those of Tiong et al. (2015) where a larger number of ZIPs were characterized. We next investigated whether any of the barley *bZIPs* would respond to Zn‐deficiency. Of those investigated, *HvbZIP1* and *HvbZIP10* were the most markedly upregulated by Zn deficiency, showing a response in both root and shoot (Figure 8). *HvbZIP58* was moderately upregulated in root and shoot whereas *HvbZIP57* was upregulated only in root. *HvbZIP56* and *HvbZIP62* showed little change after 7 days Zn deficiency and in fact Hv*bZIP56* was down‐regulated after 14 days in the shoot. We also checked whether *HvbZIP56* and *HvbZIP62* were upregulated over a shorter time period (3–5 days) in case these were early‐regulated genes; there were no marked effects on expression over this shorter time period (results not shown).

### 3.7 Investigating the presence of ZDRE motifs in promoters of barley ZIPs and bZIPs

Bioinformatic analysis was conducted to identify the promoter regions for barley *ZIP* transporter genes and then these were analysed for the presence of ZDRE motifs or the nearest similar sequence and whether palindromic in nature and containing a TCGA core. Only *HvZIPs 3, 5, 7, 8, 10* contain palindromic ZDRE motifs in their promoters (Table S6) and these have all been shown to be upregulated under Zn deficiency (Tiong et al., 2015; Figure S12). The others do not have this motif (the closest sequence is listed in Table S6) and, apart from *HvZIP13*, these ZIPs have not been shown to have a clear role under Zn deficiency. We also identified the promoters for the barley bZIPs and, where these were available, we analysed them for the presence of ZDRE domains. The only ones with a completely conserved ZDRE domain, where promoter information was available, were *HvbZIP1* and *HvbZIP5*8.

### 3.8 Subcellular localization of HvbZIP56

**Figure 9:**
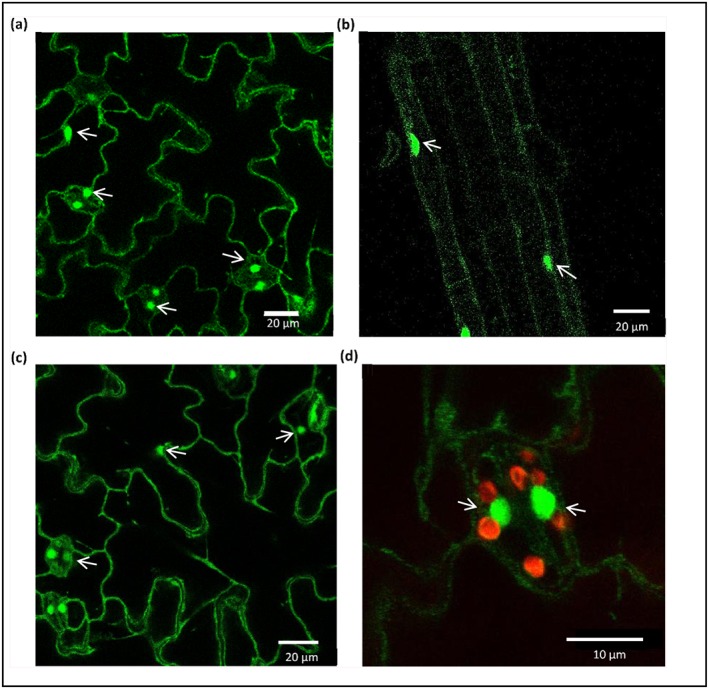
Subcellular localization of HvbZIP56‐GFP. Confocal microscopy showing stable expression of *35S::HvbZIP56‐GFP* in Arabidopsis *bzip19‐4 bzip23‐2* mutant displaying expression in cytoplasm and nuclei (white arrows) of cotyledon cells (a) and root cells (b). The expression is seen in the nuclei of both epidermal cells and guard cells and in cytoplasmic strands (c). At higher magnification the expression of HvbZIP56 in the nucleus (green) of guard cells is seen with the chloroplast autofluorescence shown in red (d)

Confocal imaging of HvbZIP56‐GFP expressed transiently in tobacco or stably in Arabidopsis showed HvbZIP56 localizing to the cytoplasm and nucleus (Figure 9, Figure S13 and Movie S1). Colocalization with the nucleus using Hoechst for nuclear staining is shown in the *HvbZIP56*‐expressing Arabidopsis lines (Figure S13ii). There was no marked difference in localization when seedlings were grown with or without Zn and in both cases HvbZIP56‐GFP was seen at the nucleus and cytoplasm. In tobacco cells, we could distinguish the plasma membrane using LTI6b‐mOrange2 (McGavin, Mitchell, Cock, Wright, & MacFarlane, 2012) and HvbZIP56 was localized adjacent to this, supporting a cytoplasmic localization (Figure S13i). The Movie S1, showing HvbZIP‐GFP expression in the Arabidopsis double mutants, supports this cytoplasmic localization as cytoplasmic strands can be seen and again the localization of HvbZIP56‐GFP in the guard cell nuclei is clearly evident.

## 4 DISCUSSION

This study provides new insight into the regulatory mechanisms controlling Zn homeostasis in cereals and extends what is known about the systems regulating Zn uptake, transport and partitioning in Arabidopsis. An important goal was to identify bZIP transcription factors in barley that may play a role in Zn‐regulatory processes. From our bioinformatics analysis, we identified seven related F‐group bZIPs (*HvbZIP1*, *10, 55, 56, 57, 58, 62*), and demonstrated that they were all expressed in Golden Promise. This group showed around 30% or higher identity to each other with some members (HvbZIP1 and HvbZIP58) showing up to 73% identity. Although an additional sequence, AK361769.1, had previously been included as a barley F‐group member named *HvbZIP61* (Pourabed et al., 2015), our analysis suggests that instead it is more likely to be an aphid sequence. HvbZIP56 and HvbZIP62, which are 65% identical, show the closest percentage identity to AtbZIP19 and AtbZIP23 (41–45%). All the barley F‐group bZIPs have a highly conserved bZIP domain but diverge somewhat after this region. In addition, all contain the Cys/His rich domains 1 and 2 except *HvbZIP10*. *HvbZIP1* and *HvbZIP58*, the most closely related barley F group, are slightly shorter proteins than the rest.

Phylogenetic analysis shows that most bZIP groups contain monocot and dicot members suggesting that diversification of bZIPs occurred before the divergence of monocots and dicots (Liu & Chu, 2015; Pourabed et al., 2015). Our analysis for the F group concurs with this but shows that while Arabidopsis contains only three F‐group bZIPs, barley has seven. This indicates that gene duplication and further diversification occurred subsequent to the dicot/monocot divergence. We set out to define more clearly the role of these F‐group bZIP transcription factors in cereals and to test whether there was a link with the Zn‐deficiency response mechanism.

### 4.1 Functional complementation demonstrates a role for barley bZIPs in Zn‐deficiency responses

The availability of genetic resources in barley is not as comprehensive as in Arabidopsis and therefore in this study we took advantage of a heterologous complementation approach using Arabidopsis *bzip19 bzip23* double mutants to test whether barley bZIPs could restore Zn‐deficiency responses. Five *bzip19 bzip23* mutants having different combination of T‐DNA insertion sites were isolated in this study, four of which were unique. All were knockout mutants as no full‐length transcripts were detected. When grown under 0.5 MS, which is routinely used for mutant analysis, all *bzip19 bzip23* double mutants showed extreme hypersensitivity to Zn‐deficient conditions. This growth defect in the *bzip19‐4 bzip23‐2* double mutant could be rescued by as low as 1 μm Zn. We demonstrated that this hypersensitive phenotype was specific to Zn‐deficient conditions as other micronutrient deficiencies (Mn, Fe and Cu) had no specific effect on the *bzip19 bzip23* mutant in comparison to wild type. This confirms a role for the transcription factors AtbZIP19 and AtbZIP23 in modulating the response of Arabidopsis to low Zn exposure. The *bzip19* single mutants also displayed sensitivity to Zn deficiency although this was less severe than the response seen in the double mutant. No significant differences were seen in the *bzip23* mutants. Using an alternative medium, MRGL, a Zn‐deficiency phenotype has been observed for *bzip19* and, less so, *bzip23* single mutants (Inaba et al., 2015). We used 0.5 MS to investigate complementation by barley bZIPs as we observed a clear phenotype for the double mutants when Zn was omitted.

The barley F‐group bZIP transcription factors, HvbZIP56 and HvbZIP62, were shown to function in the Zn‐deficiency response by demonstrating their ability to restore growth of the Arabidopsis *bzip19‐4 bzip23‐2* double mutant almost to wild‐type levels when grown in Zn‐deficient media. Moreover, these F‐group barley bZIPs were able to convey Zn‐deficiency induced expression of particular Arabidopsis ZIP transporter genes to the double mutant. This suggests that the regulation of ZIP transporter expression by F‐group bZIPs in response to Zn deficiency may be conserved across species. Additionally, we demonstrated that HvbZIP56 restored *AtNAS4* and *AtDEFL203* expression and HvbZIP62 restored *AtNAS4* expression in Arabidopsis *bzip19 bzip23* double mutants under Zn deficiency, again suggesting a conserved action.

Assunção et al. (2013) showed that *AtbZIP1*9 or *AtbZIP23* expression (under the 35S promoter) in the double mutant line background (*bzip19‐1 bzip23‐1*) completely complements the Zn‐hypersensitive phenotype suggesting that *bZIP19* and *bZIP23* are redundant and thus function as homodimers. However, as *HvbZIP56* or *HvbZIP62* (also expressed under the 35S promoter) do not fully complement the double, then perhaps there are certain functions carried out by the Arabidopsis bZIPs that cannot be performed as effectively by the barley bZIPs. Perhaps each *HvbZIP* expressed alone may not regulate exactly the same cohort of genes or to the same level as AtbZIP19 and AtbZIP23. Having an expanded number of bZIPs, it may be the case that barley bZIPs function more efficiently as heterodimers to regulate certain genes and so it may be useful in the future to test whether expressing two barley bZIPs together fully rescues the double mutant. It will also be important to test the other barley bZIPs to determine whether they all play a role in Zn deficiency or whether there is functional diversity within this group. In Arabidopsis, the third F‐group member, AtbZIP24, has been implicated in resistance to salt stress and it is therefore possible that some of the barley F‐group bZIPs have broader roles. However, none of the barley bZIPs isolated here show a stronger percentage identity to AtbZIP24 than they do to AtbZIP19 or AtbZIP23.

When grown in Zn‐deficient media, all lines tested showed very low Zn levels, but additionally the double mutant showed a lower Fe content compared to wild type. This was restored in the *HvbZIP56*‐expressing lines and so the incomplete rescue of the growth phenotype was not explained by differing metal micronutrient levels. The cause of this Fe imbalance in the double mutant is not certain but a number of the genes regulated by AtbZIP19 and AtbZIP23 (e.g., *AtNAS2* and *AtNAS4*) have also been implicated in Fe distribution and homeostasis (Assunção et al., 2010). Therefore, differences in the expression of these genes may result in metal imbalances. It should be noted that the stunted growth phenotype is specific to low Zn exposure as only when grown on low Zn were the double mutants significantly different from wild type. However, the differences in Fe content in the double mutant seen under low Zn exposure in combination with low Zn could contribute to the stunted phenotype.

### 4.2 The presence of ZDRE motifs in the promoter of barley ZIP transporters supports a conserved mechanism in responding to Zn deficiency

In addition to Arabidopsis thaliana, conserved ZDRE domains have now been found in ZIP4 orthologues from four other species of Brassicaceae (Lin, Hassan, Talukdar, Schat, & Aarts, 2016). Two ZDRE domains were found and certainly for *Arabidopsis* thaliana and *Noccaea caerulescens*, these ZDRE domains do seem to play important roles in the response of AtZIP4/NcZNT1 to Zn deficiency. For all these species, they are located within 250bp of the predicted ATG start codon of the gene and all with similar distance between both ZDREs. If the mechanism for responding to Zn deficiency is conserved in cereals, we reasoned that the barley ZIPs that are upregulated by Zn deficiency may contain a ZDRE domain. We show in our study that *HvZIP5*, *HvZIP10*, and *HvZIP13* are upregulated by Zn deficiency, whereas *ZIP14* is not. This is in agreement with Tiong et al. (2015) who analysed 13 *HvZIP* genes and found that six (*HvZIP3*, *HvZIP5*, *HvZIP7*, *HvZIP8*, *HvZIP10*, and *HvZIP13*) of the 13 analysed were strongly induced by Zn deficiency, whereas the remaining seven (*HvIRT1*, *HvZIP1*, *HvZIP2*, *HvZIP6*, *HvZIP11*, *HvZIP14*, and *HvZIP16*) were not. Our analysis of the promoter regions for all of these shows that the genes that were enhanced by Zn deficiency contain a palindromic ZDRE domain. The only exception to this is *HvZIP13*, where the closest motif was only 1bp  different and not palindromic; it remains to be tested whether this is a motif important in responding to Zn deficiency or whether *HvbZIP13* is regulated by an alternative mechanism. Overall, our results in barley demonstrate the presence of a ZDRE domain in the promoters of transporter genes strongly induced by low Zn; this suggests a conserved mechanism in responding to Zn deficiency.

### 4.3 Regulation of F‐group bZIPs

In Arabidopsis, *AtbZIP19* and *AtbZIP23* were initially reported as being approximately twofold upregulated under Zn deficiency, whereas *At* *bZIP24*, another F‐group member, was unaffected by Zn status (Assunção et al., 2010). In a later review, the same group suggest that in a detailed analysis, *AtbZIP19* and *AtbZIP23* expression levels are not significantly affected under Zn deficiency (Assunção et al., 2013). They hypothesized instead that a Cys/His‐motif upstream of the basic region may function as a Zn‐sensor and postulated a model, whereby this region acts in a post‐translational‐regulation mechanism. Under normal Zn supply, it was suggested that Zn would be bound to this motif, and in this conformation, the transcription factor would be nonfunctional; when released from this motif under Zn deficiency, they would become active and would target the promoters of ZIP transporters in the nucleus. Concerning transcriptional regulation, Inaba et al. (2015) claimed that *AtbZIP19* and *AtbZIP23* do not respond to Zn deficiency but did in fact show mild upregulation of *AtbZIP19* under these conditions compared to basal conditions. In barley, under Zn deficiency, we found that *HvbZIP1*, *HvbZIP10*, and *HvbZIP58* were significantly upregulated in roots and shoots; *HvbZIP5*7 showed up‐regulation only in root tissue, whereas no induction was observed for *HvbZIP56* and *HvbZIP62*. The mechanism whereby these transcription factors are activated in response to Zn deficiency is therefore still to be determined. Targeting from the cytoplasm to the nucleus as part of the mechanism for activation of F‐group bZIPs has been supported for other bZIPs. For example, BdbZIP10/11 showed enhanced levels in the nucleus following exposure to oxidative stress, and this was suggested to be due to post‐translational modifications enhancing stability or directing subcellular localization (Glover‐Cutter et al., 2014). Yang et al. (2009) also found that AtbZIP24 was re‐distributed preferentially to the nucleus in response to salt stress. Our study with HvbZIP56 and the study with AtbZIP19 and AtbZIP23 (Inaba et al., 2015) indicates that these F‐group bZIPs are present in the nucleus during Zn sufficiency as well as in Zn deficiency. Thus, while this is consistent with them acting as transcription factors to upregulate *ZIP* transporter genes, redistribution to the nucleus solely under Zn deficiency cannot explain the mechanism for Zn responsiveness. Further work is necessary to completely understand the mechanism by which the F‐group bZIPs respond in adapting to Zn deficiency.

## 5 CONCLUSIONS

Zn deficiency is a global problem and is arguably one of the leading micronutritional restraints to crops, leading to poor agricultural yields and lowered nutritional quality (Cakmak, 2008). A greater understanding of the molecular mechanism regulating the adaption of plants to insufficient Zn is an important step forward in developing biofortification strategies, helping to improve human nutrition and alleviate health problems associated with Zn deficiency. In plants, there is an intricate network regulating responses to low Zn. Our findings indicate that particular members of the barley F‐group bZIPs can function in adapting to Zn deficiency and therefore could represent novel targets for improving nutrient efficiency in crops.

## Supporting information

Movie S1 Supporting information

Fig. S1. Phylogenetic analysis of barley F group bZIPs and BAJ92973/HvbZIP61.
Figure S2 Amino acid multiple alignment of Arabidopsis and barley F group bZIPs.
Figure S3 Mutant alleles for At*bZIP19* and At*bZIP23*.
Figure S4 Zn‐deficiency phenotype of the additional *bzip19 bzip23* double mutants in plate and hydroponic assays.
Figure S5 Direct comparison of single and double mutant bZIP alleles.
Figure S6 Cu deficiency (i) or Fe deficiency (ii) does not specifically affect *bzip19, bzip23* and *bzip19 bzip23* mutants compared to wild type.
Figure S7 Metal concentrations in wild type (WT), *bzip19‐4 bzip23‐2* double mutant, and bZIP56‐expressing double mutant lines.
Figure S8 HvbZIP56‐expressing *bzip19‐4 bzip23‐2* lines grow to a similar level to *bzip19‐4* single mutant under Zn‐deficiency.
Figure S9 HvbZIP56 expression in wild type Arabidopsis does not influence their response to Zn deficiency or Zn excess.
Figure S10. HvbZIP62 restores gene expression in response to Zn‐deficiency in the Arabidopsis *bzip19‐4 bzip23‐2* mutant.
Figure S11 Barley displays deficiency symptoms when grown under Zn‐deficient conditions.
Figure S12 Regulation of ZIPs following imposition of Zn‐deficiency conditions.
Figure S13 Localisation of HvbZIP56‐GFP in tobacco (i).
Table S1 Primer purpose, nomenclature and sequence
Table S2 HvbZIPs identified from bioinformatics analysis
Table S3. Accession numbers for sequences used in the phylogenetic analysis in Figure 1.
Table S4 Percentage identity/similarity of Arabidopsis and barley F group bZIP
Table S5 Nomenclature of mutant lines
Table 6 ZDRE motifs and position
