# Supplementary material for: F‐group bZIPs in barley—a role in Zn deficiency
Source: Plant Cell Environ. 2017 Sep 19;40(11):2754–70. doi: 10.1111/pce.13045 (PMC5656896; doi:10.1111/pce.13045)
Supplement: Supplementary file 2 — Fig. S1. Phylogenetic analysis of barley F group bZIPs and BAJ92973/HvbZIP61. Figure S2 Amino acid multiple alignment of Arabidopsis and barley F group bZIPs. Figure S3 Mutant alleles for AtbZIP19 and AtbZIP23. Figure S4 Zn‐deficiency phenotype of the additional bzip19 bzip23 double mutants in plate and hydroponic assays. Figure S5 Direct comparison of single and double mutant bZIP alleles. Figure S6 Cu deficiency (i) or Fe deficiency (ii) does not specifically affect bzip19, bzip23 and bzip19 bzip23 mutants compared to wild type. Figure S7 Metal concentrations in wild type (WT), bzip19‐4 bzip23‐2 double mutant, and bZIP56‐expressing double mutant lines. Figure S8 HvbZIP56‐expressing bzip19‐4 bzip23‐2 lines grow to a similar level to bzip19‐4 single mutant under Zn‐deficiency. Figure S9 HvbZIP56 expression in wild type Arabidopsis does not influence their response to Zn deficiency or Zn excess. Figure S10. HvbZIP62 restores gene expression in response to Zn‐deficiency in the Arabidopsis bzip19‐4 bzip23‐2 mutant. Figure S11 Barley displays deficiency symptoms when grown under Zn‐deficient conditions. Figure S12 Regulation of ZIPs following imposition of Zn‐deficiency conditions. Figure S13 Localisation of HvbZIP56‐GFP in tobacco (i). Table S1 Primer purpose, nomenclature and sequence Table S2 HvbZIPs identified from bioinformatics analysis Table S3. Accession numbers for sequences used in the phylogenetic analysis in Figure 1. Table S4 Percentage identity/similarity of Arabidopsis and barley F group bZIP Table S5 Nomenclature of mutant lines Table 6 ZDRE motifs and position [file PCE-40-2754-s002.pdf]

Supporting Information

Article title: F-group bZIPs in barley – a role in Zn deficiency

Authors: **Ahmad Zulhilmi Nazri, Jonathan H.C. Griffin, Kerry A. Peaston, Douglas G.A. Alexander-Webber and Lorraine E. Williams**

The following Supporting Information is available for this article: Supporting Information Figures S1-S13 and Tables S1-S6

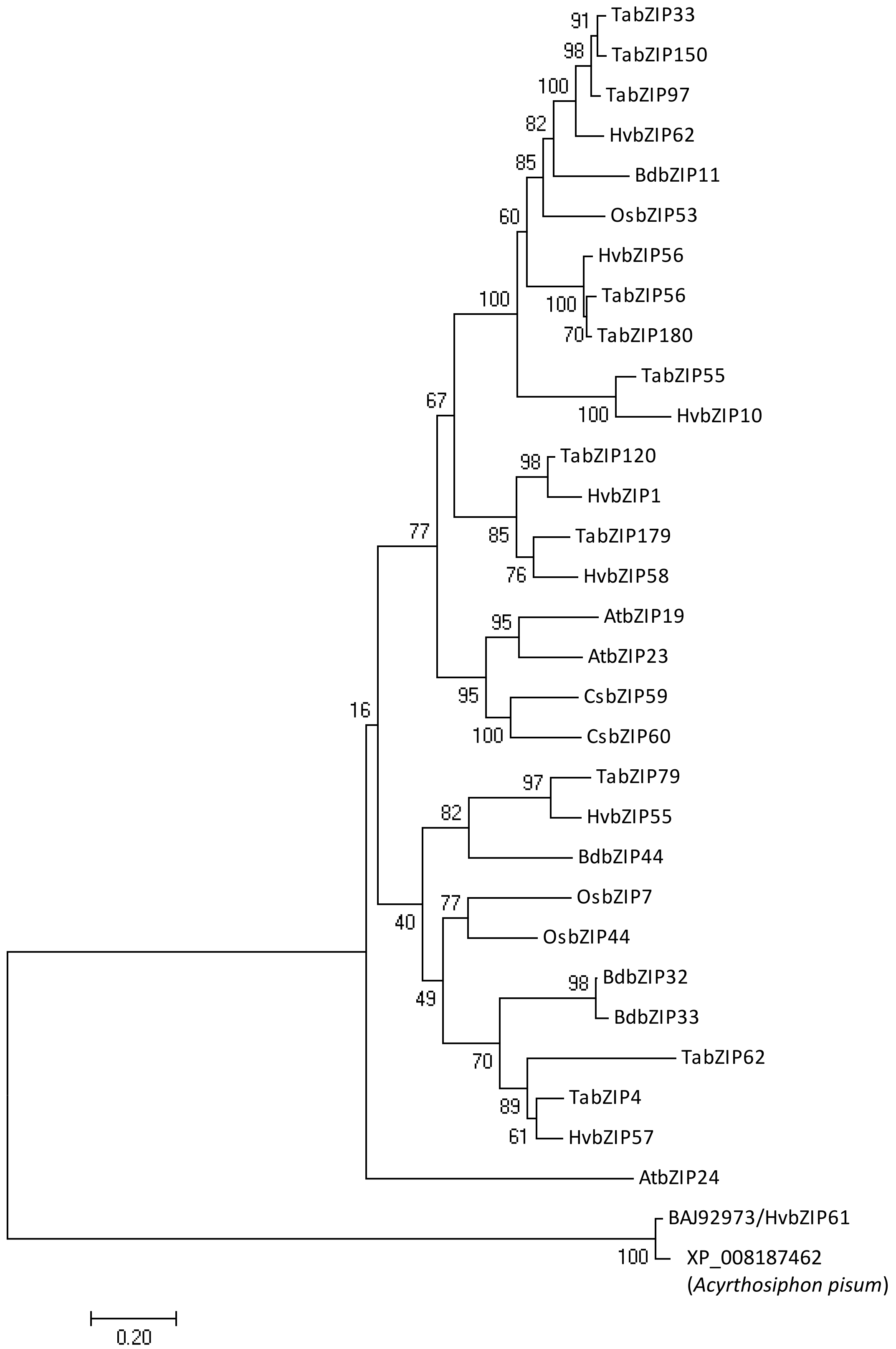
TabZIP33
TabZIP150
TabZIP97
HvbZIP62
BdbZIP11
OsbZIP53
HvbZIP56
TabZIP56
TabZIP180
TabZIP55
HvbZIP10
TabZIP120
HvbZIP1
TabZIP179
HvbZIP58
AtbZIP19
AtbZIP23
CsbZIP59
CsbZIP60
TabZIP79
HvbZIP55
BdbZIP44
OsbZIP7
OsbZIP44
BdbZIP32
BdbZIP33
TabZIP62
TabZIP4
HvbZIP57
AtbZIP24
BAJ92973/HvbZIP61
XP_008187462
(*Acyrthosiphon pisum*)
91
98
100
82
85
60
100
70
100
67
100
98
77
85
76
95
95
16
100
97
82
77
40
98
49
70
89
61
100
0.20

Fig. S1. Phylogenetic analysis of barley F group bZIPs and BAJ92973/HvbZIP61. The non-rooted, bootstrapped plot was constructed using MEGA 7 (Kumar *et al*., 2016) with a multiple alignment of bZIPs from *Hordeum vulgare, Triticum aestivum*, *Brachypodium distachyon*, *Oryza sativa*, *Arabidopsis thaliana,* and *Cucumis sativus*. As a comparison, sequence BAJ92973, originally described as HvbZIP61 and XP_008187462 from *Acyrthosiphon pisum*, are also included in the multiple alignment. The percentage of replicate trees in which the associated taxa clustered together in the bootstrap test (1000 replicates) are shown next to the branches (Felsenstein 1985). The tree is drawn to scale, with branch lengths in the same units as those of the evolutionary distances used to infer the phylogenetic tree. The evolutionary distances were computed using the Poisson correction method (Zuckerkandl and Pauling 1965) and are in the units of the number of amino acid substitutions per site. All positions containing gaps and missing data were eliminated. Accession numbers and identifier of the predicted proteins are listed in Table S3.

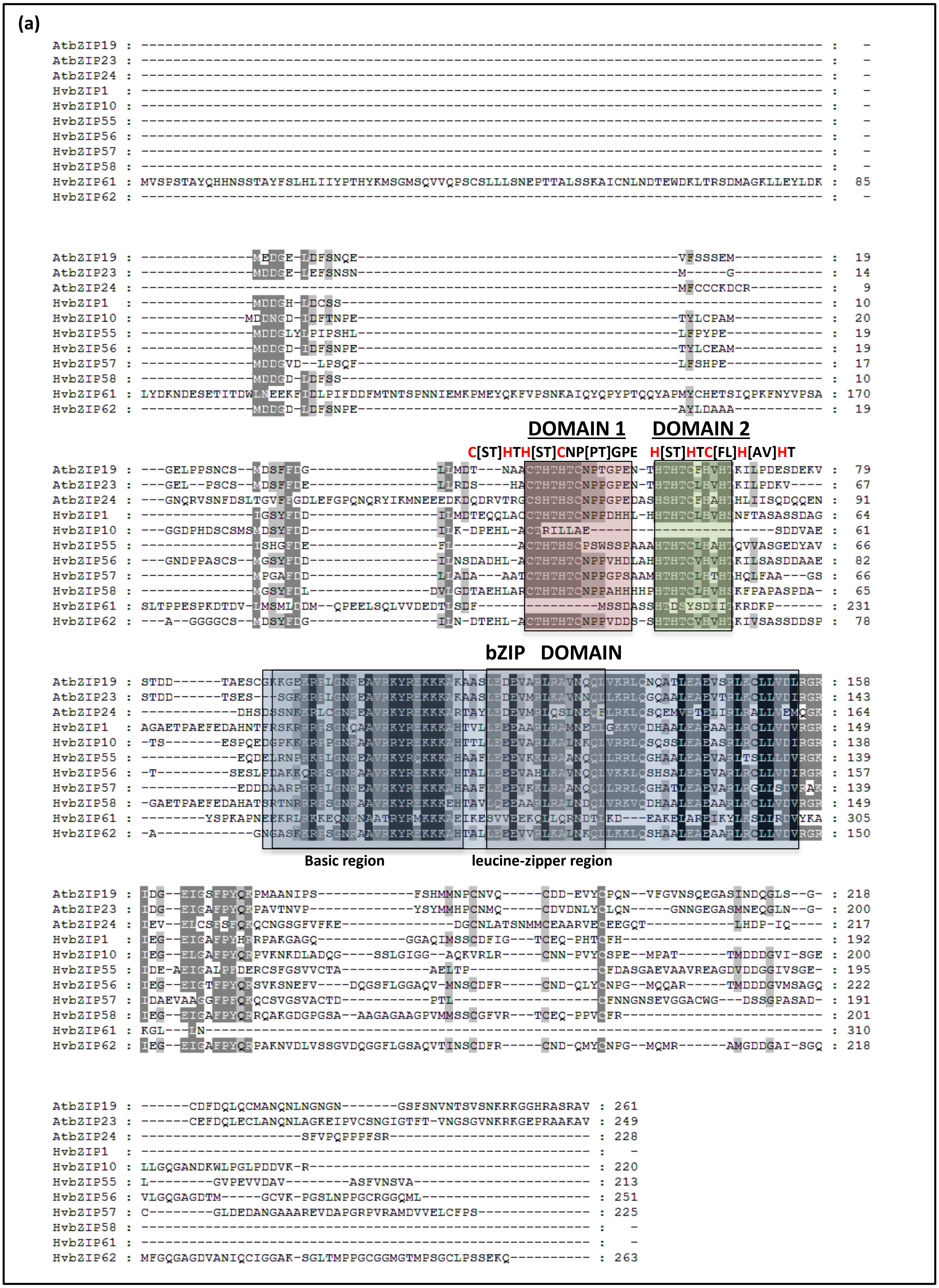
(a)
AtbZIP19
AtbZIP23
AtbZIP24
HvbZIP1
HvbZIP10
HvbZIP55
HvbZIP56
HvbZIP57
HvbZIP58
HvbZIP61
HvbZIP62
DOMAIN 1
DOMAIN 2
C[ST]HTH[ST]CNP[PT]GPE
H[ST]HTC[FL]H[AV]HT
bZIP DOMAIN
Basic region
leucine-zipper region

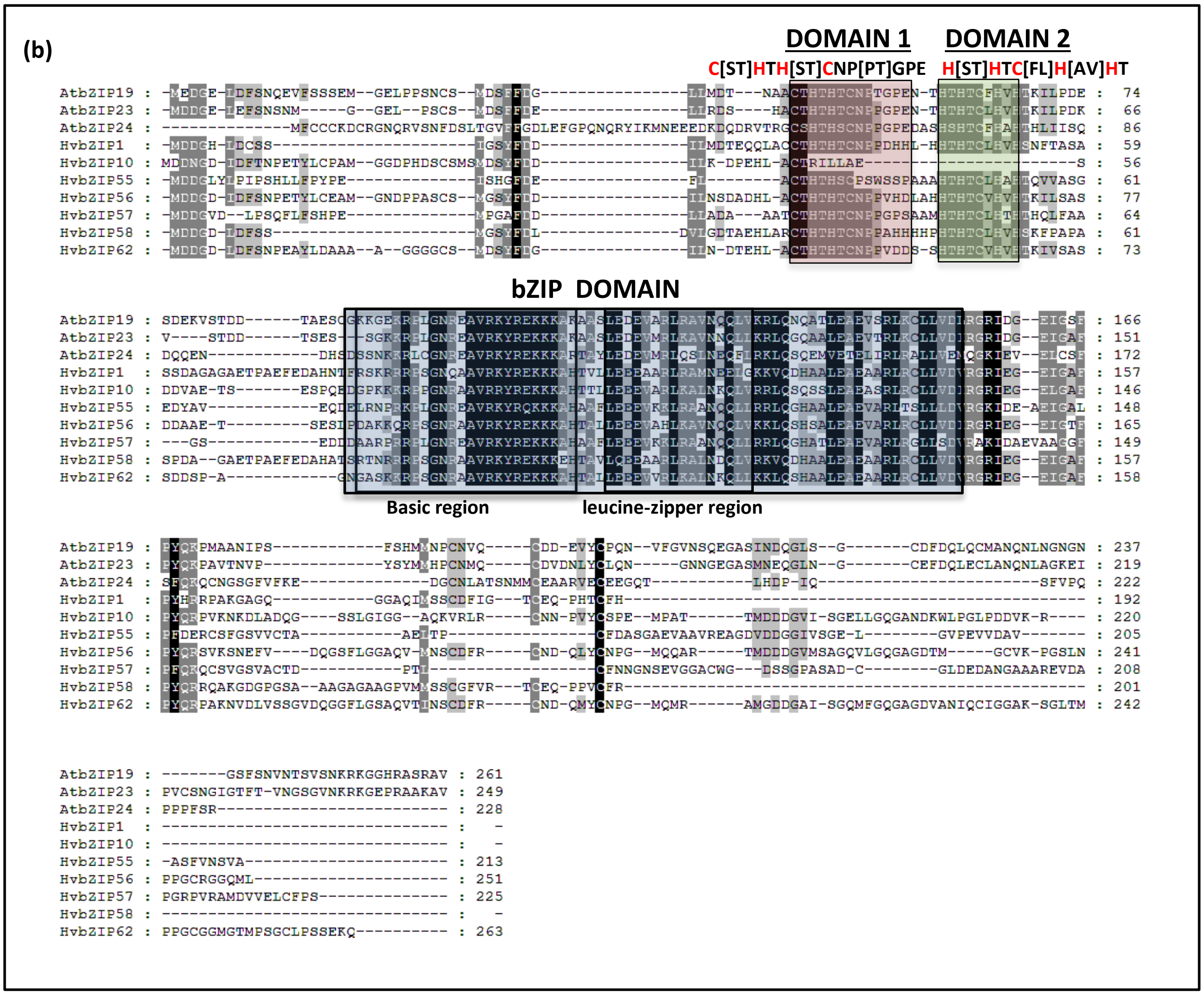

Fig. S2 Amino acid multiple alignment of Arabidopsis and barley F group bZIPs. Alignment including (a) or omitting (b) HvbZIP61/BAJ92973; a putative aphid sequence. Sequences were aligned using the Clustal Omega algorithm (Sievers et al., 2011) and presented using GeneDoc (Nicholas and Nicholas Jr, 1997). For the sequences aligned here: black = conserved residues, dark grey = conserved in at least nine of the sequences, and light grey = conserved in at least seven of the sequences. The bZIP domain and the two conserved Cys/His-rich domains (domain 1 and 2) are highlighted. Above the latter two are the sequence of the Arabidopsis F group bZIPs (Jakoby et al. 2002). The bZIP domain is boxed according to information given for AtbZIP19 at http://www.uniprot.org/ with the first box indicating the basic region and the second box indicating the leucine zipper region within the domain.

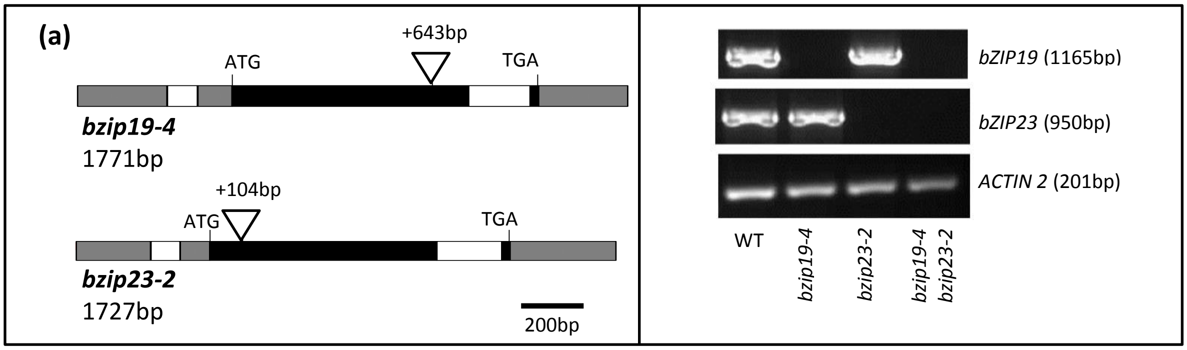
(a)
+643bp
ATG
TGA
bzip19-4
1771bp
+104bp
ATG
TGA
bzip23-2
1727bp
200bp
bZIP19 (1165bp)
bZIP23 (950bp)
ACTIN 2 (201bp)
WT
bzip19-4
bzip23-2
bzip19-4 bzip23-2

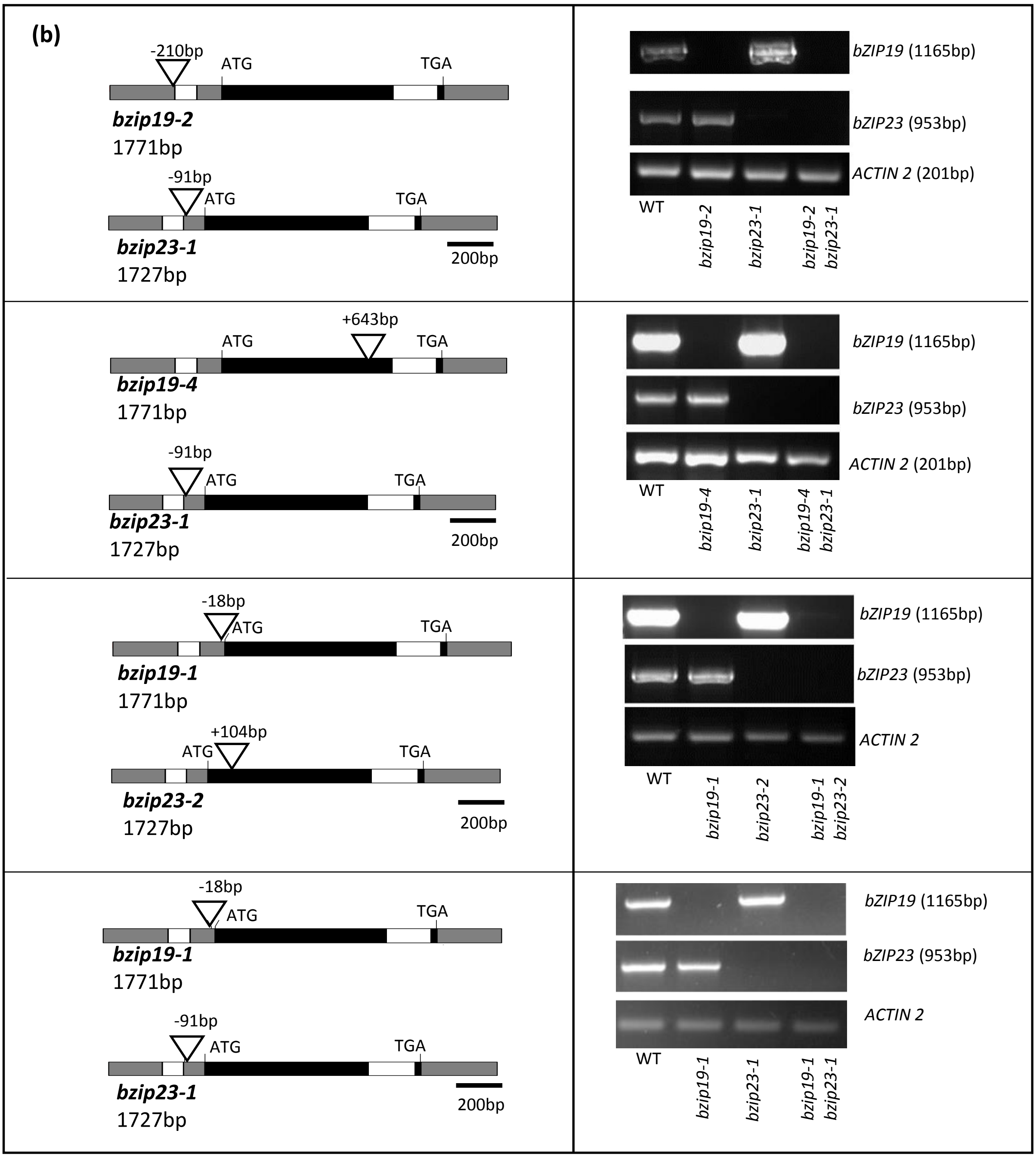

Fig. S3 Mutant alleles for At*bZIP19* and At*bZIP23*. Left, Schematic drawings showing position of inserts for mutant alleles (a) *bzip19-4 bzip23-2* (b) *bzip19-2 bzip23-1, bzip19-4 bzip23-1*, *bzip19-1 bzip23-2*, *bzip19-1 bzip23-1*. Black bars exons, white bars introns and grey bars untranslated regions. A of ATG is taken as 0. Triangle represents T-DNA. Right, corresponding gel image showing the expression of *AtbZIP19* and *AtbZIP23* at RNA level in the different mutant lines, with *AtACTIN2* as control.

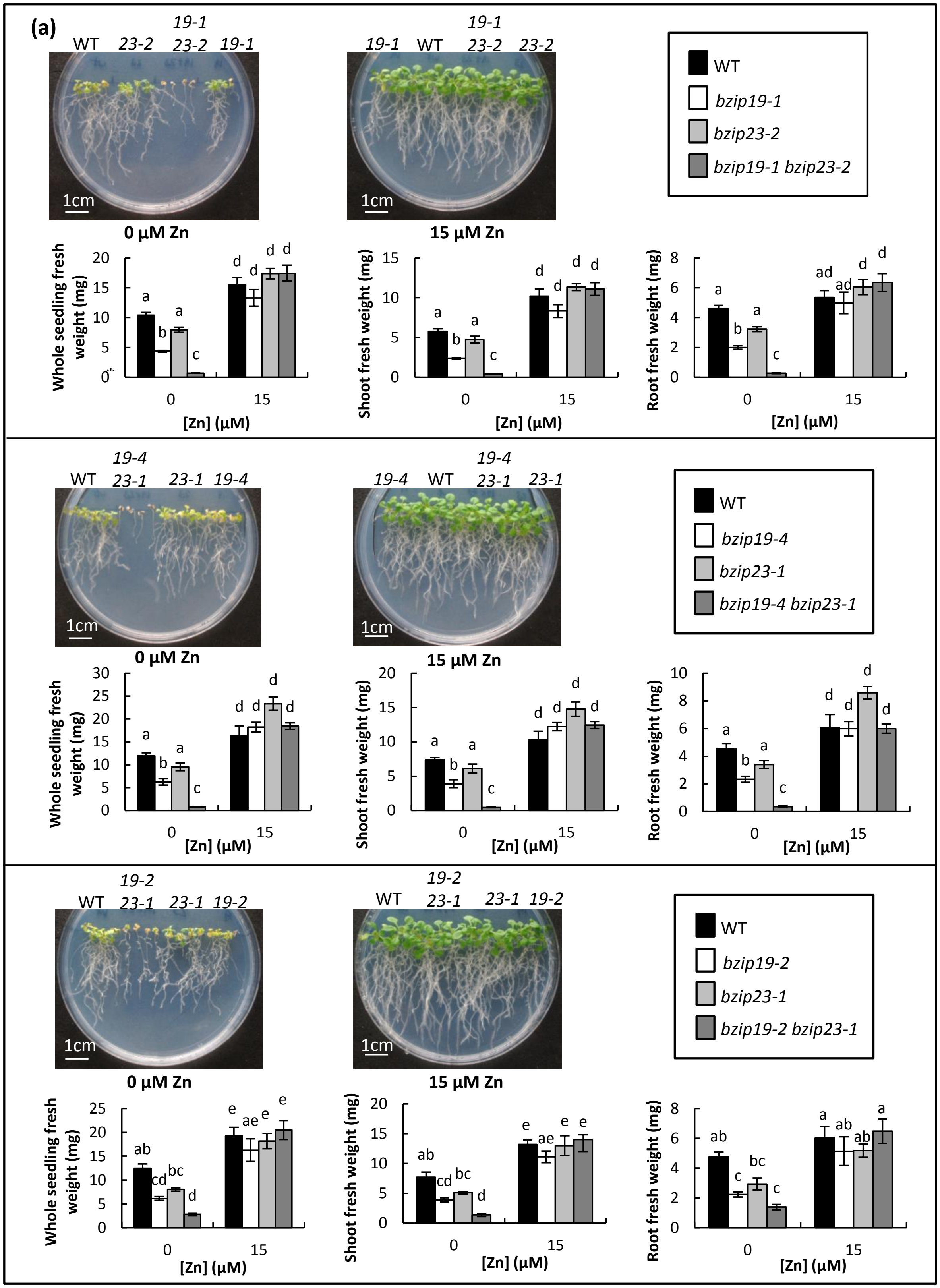
(a)
WT
23-2
19-1 23-2
19-1
0 µM Zn
15 µM Zn
1cm
bzip19-1
bzip23-2
bzip19-1 bzip23-2
Whole seedling fresh weight (mg)
Shoot fresh weight (mg)
Root fresh weight (mg)
[Zn] (µM)
19-4 23-1
23-1
19-4
bzip19-4
bzip23-1
bzip19-4 bzip23-1
19-2 23-1
19-2
bzip19-2
bzip19-2 bzip23-1

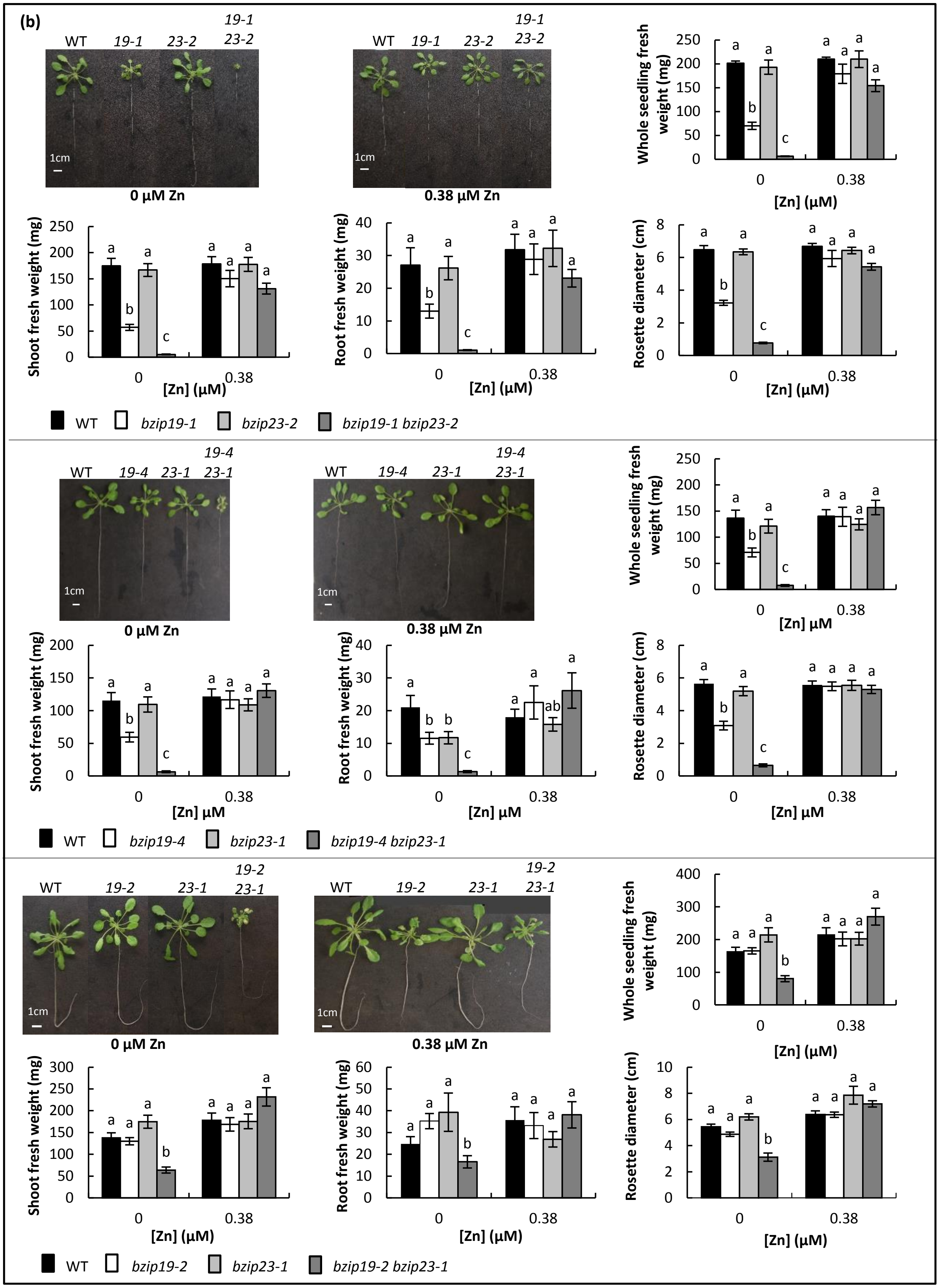
(b)
WT
19-1
23-2
19-1 23-2
1cm
0 µM Zn
0.38 µM Zn
Whole seedling fresh weight (mg)
Shoot fresh weight (mg)
Root fresh weight (mg)
Rosette diameter (cm)
[Zn] (µM)
WT
bzip19-1
bzip23-2
bzip19-1 bzip23-2
19-4
23-1
19-4 23-1
[Zn] µM
WT
bzip19-4
bzip23-1
bzip19-4 bzip23-1
19-2
23-1
19-2 23-1
WT
bzip19-2
bzip23-1
bzip19-2 bzip23-1

Fig. S4 Zn-deficiency phenotype of the additional *bzip19 bzip23* double mutants in plate and hydroponic assays. Wild type (WT), *bzip19, bzip23,* and *bzip19 bzip23* T-DNA insertion *Arabidopsis thaliana* mutants grown on agarose half MS plates for 21 days with 0 µM Zn or 15 µM Zn (a) or hydroponic culture for 40 days with 0 µM Zn or 0.38 µM Zn (b). For each growth condition, fresh weight measurements for total, shoot and root weight are shown. Rosette diameter is also shown for the hydroponically grown plants. For the plate assays, the means (+/- SEM) were based on six plates with four seedlings per line, per plate, each plate containing four plant lines. For hydroponic assays the means (+/- SEM) were based on 30 plants. For plate and hydroponic assays means not sharing a letter are significantly different ($P \leq 0.05$); Tukey post-hoc test.

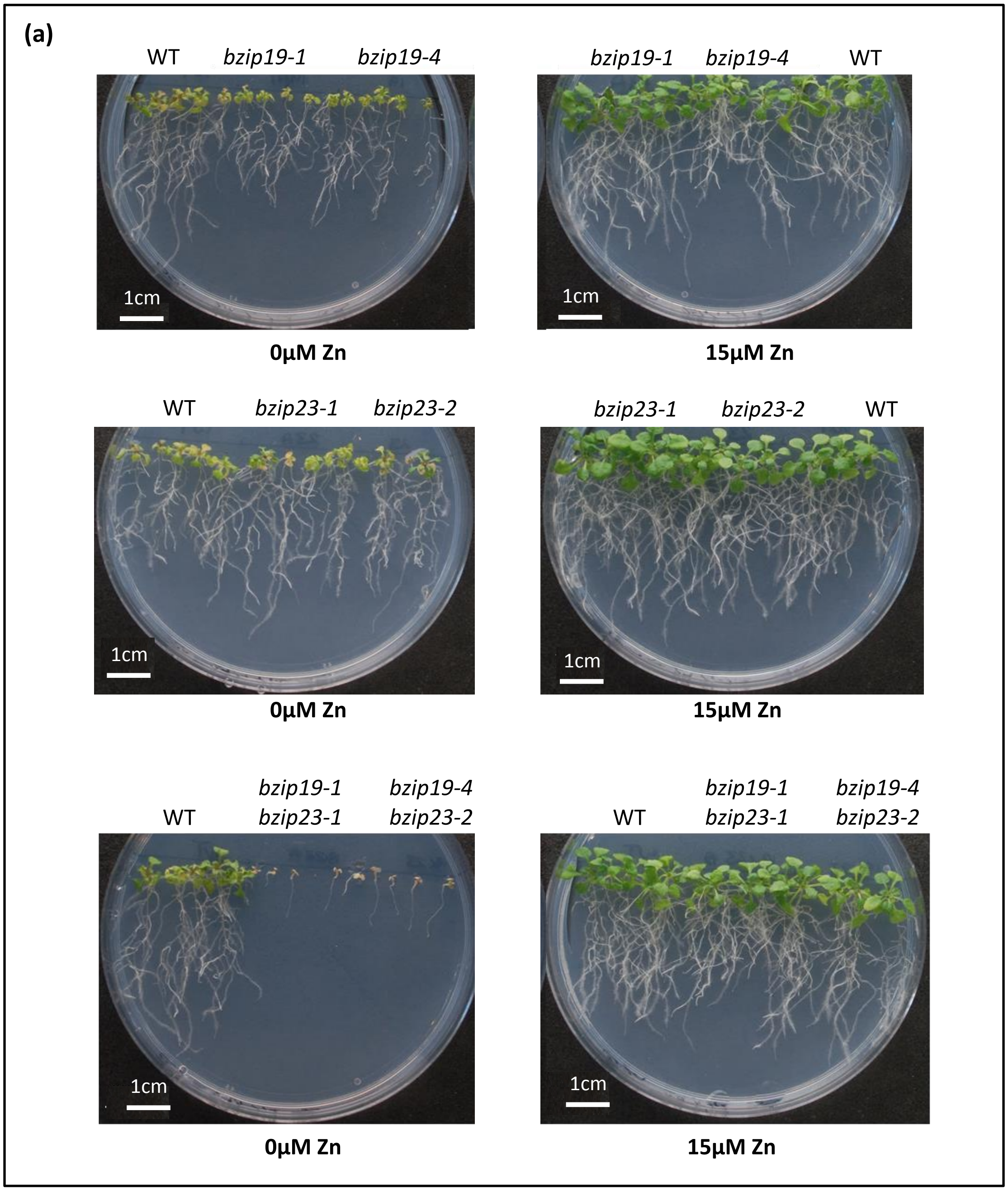
(a)
WT
bzip19-1
bzip19-4
1cm
0µM Zn
bzip19-1
bzip19-4
WT
1cm
15µM Zn
WT
bzip23-1
bzip23-2
1cm
0µM Zn
bzip23-1
bzip23-2
WT
1cm
15µM Zn
WT
bzip19-1
bzip23-1
bzip19-4
bzip23-2
1cm
0µM Zn
WT
bzip19-1
bzip23-1
bzip19-4
bzip23-2
1cm
15µM Zn

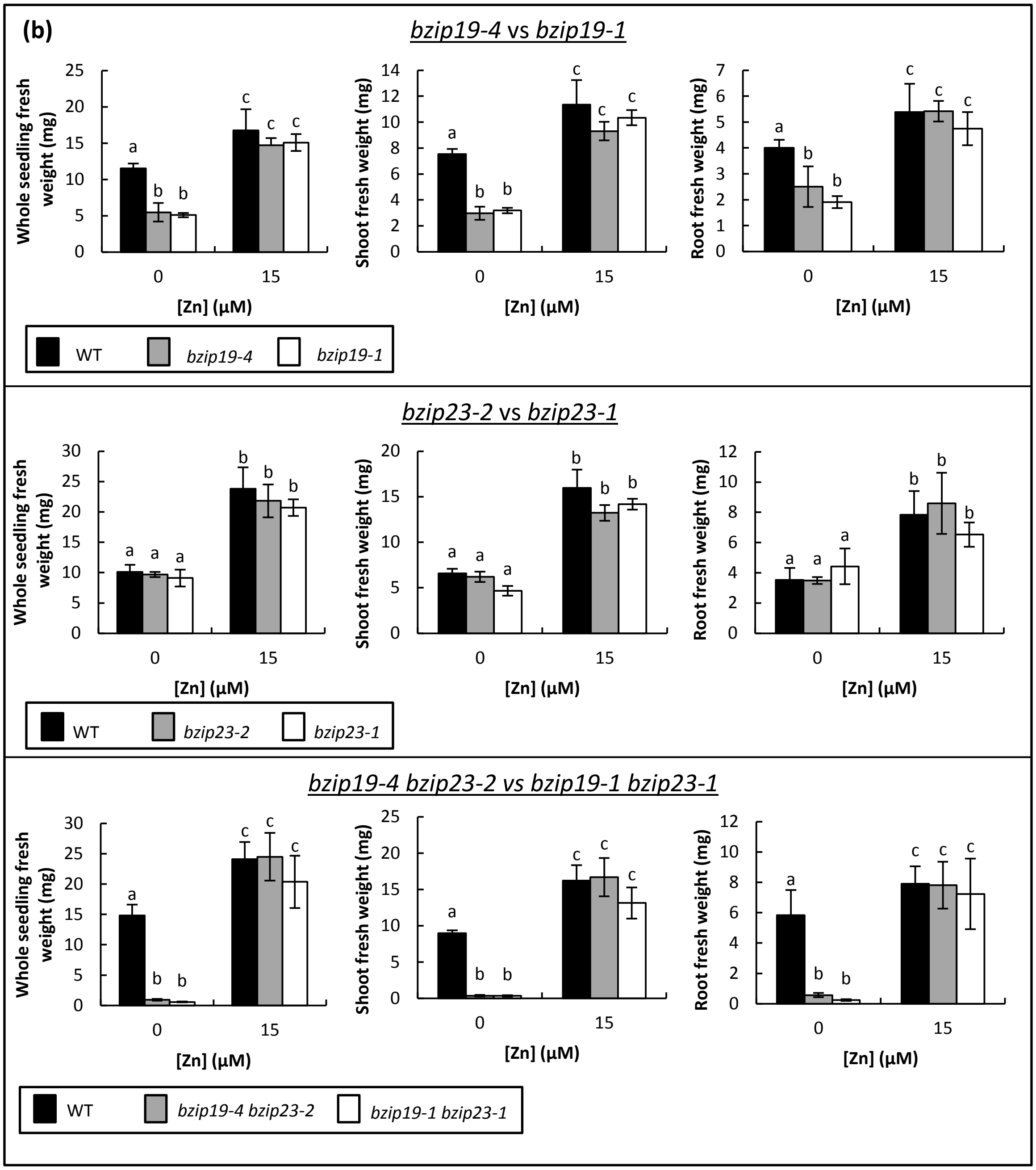

Fig. S5 Direct comparison of single and double mutant bZIP alleles. (a) *bzip19-4* and *bzip19-1, bzip23-2* and *bzip23-1, bzip19-4 bzip23-2 and bzip19-1 bzip23-1* were directly compared by growing on agarose half MS plates for 21 days with 0 µM Zn or 15 µM Zn. Wild type (WT) plants were included. (b) For each growth condition, total fresh weight is shown. The means (+/- SEM) were based on six plates, with five seedlings per line, per plate, each plate containing three plant lines. Means not sharing a letter are significantly different (*P*≤0.05); Tukey post-hoc test.

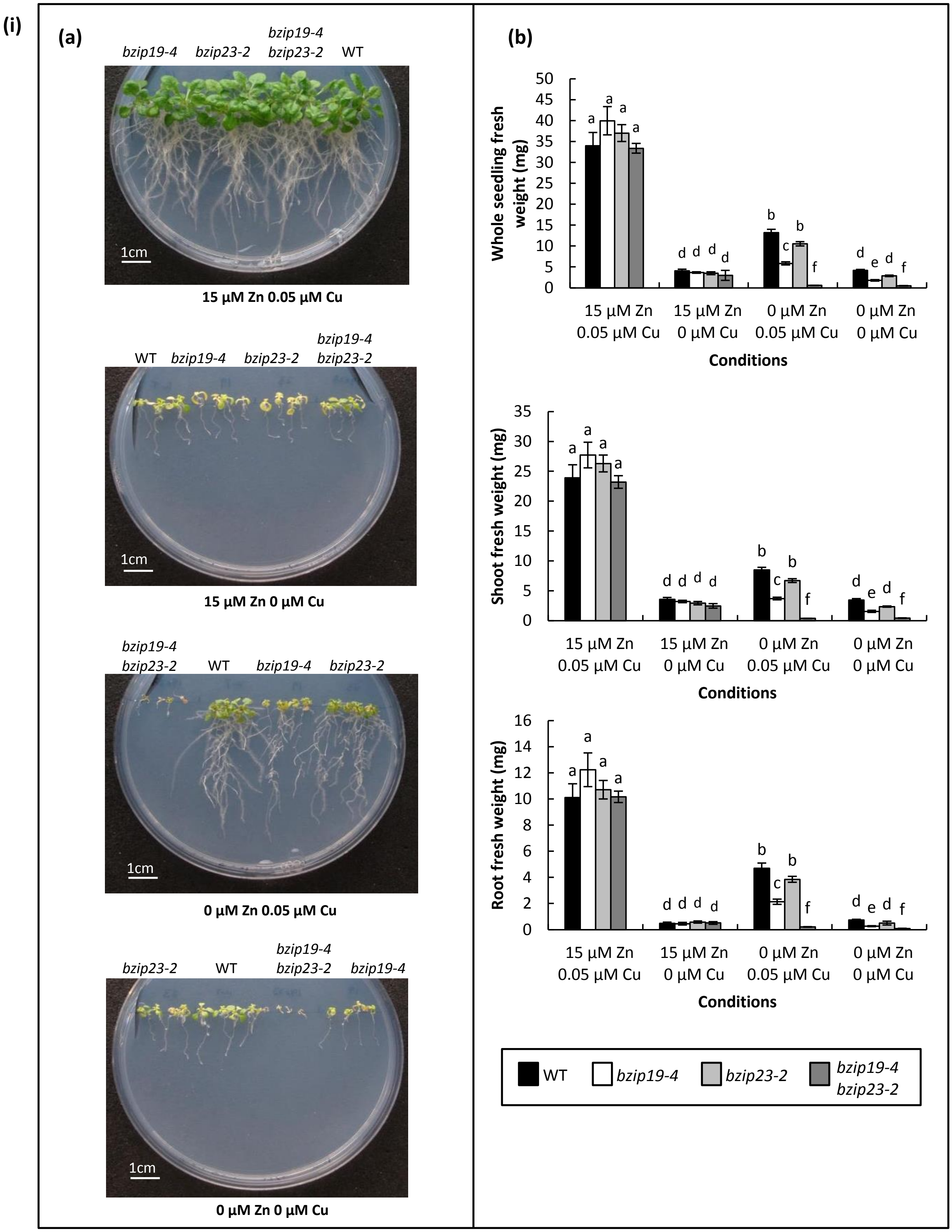
(i)
(a)
bzip19-4
bzip23-2
bzip19-4 bzip23-2
WT
1cm
15 µM Zn 0.05 µM Cu
WT
bzip19-4
bzip23-2
bzip19-4 bzip23-2
1cm
15 µM Zn 0 µM Cu
bzip19-4 bzip23-2
WT
bzip19-4
bzip23-2
1cm
0 µM Zn 0.05 µM Cu
bzip23-2
WT
bzip19-4 bzip23-2
bzip19-4
1cm
0 µM Zn 0 µM Cu
(b)
Whole seedling fresh weight (mg)
Shoot fresh weight (mg)
Root fresh weight (mg)
15 µM Zn 0.05 µM Cu
15 µM Zn 0 µM Cu
0 µM Zn 0.05 µM Cu
0 µM Zn 0 µM Cu
Conditions
WT
bzip19-4
bzip23-2
bzip19-4 bzip23-2

(ii)

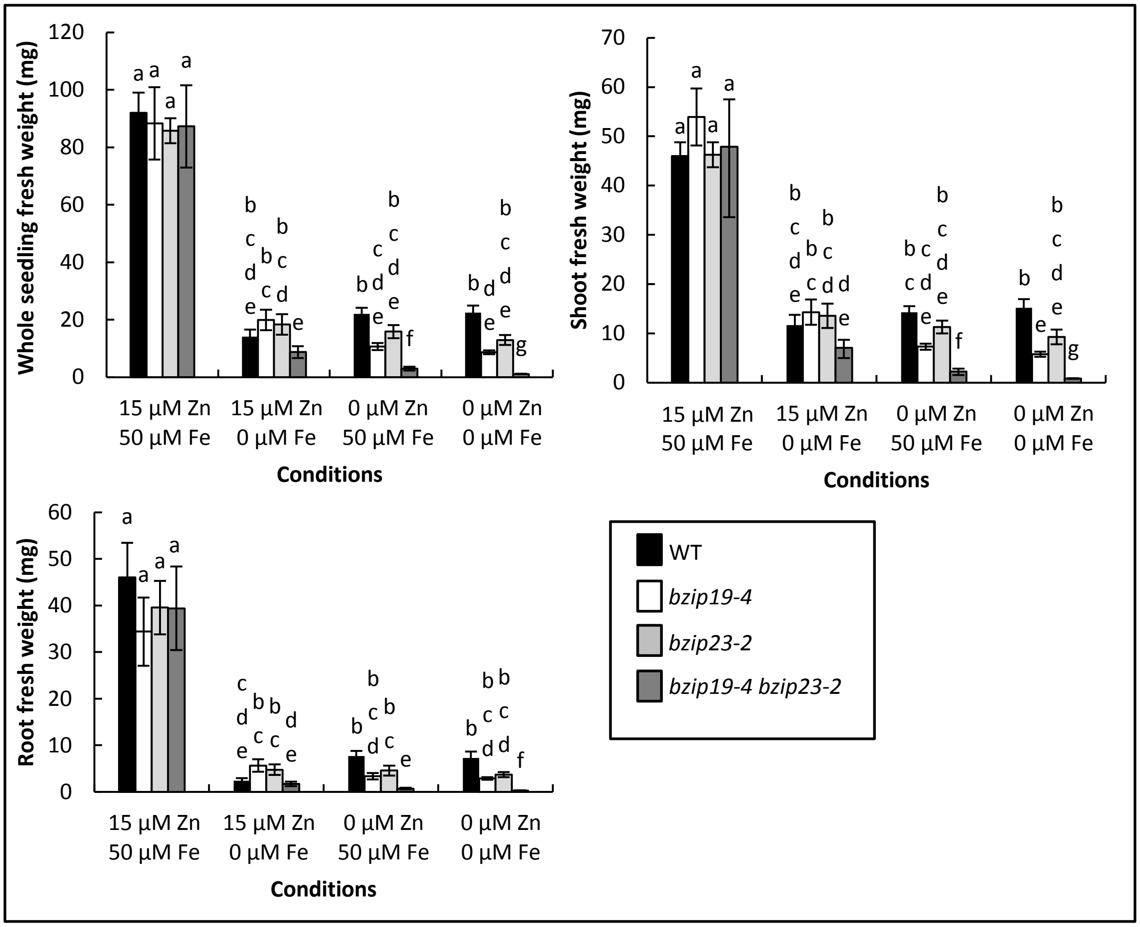

Fig. S6 Cu deficiency (i) or Fe deficiency (ii) does not specifically affect *bzip19, bzip23* and *bzip19 bzip23* mutants compared to wild type. (a) Wild type (WT), *bzip19-4, bzip23-2* and *bzip19-4 bzip23*-2 mutants grown on half MS media under a range of Cu and Zn concentrations (i) or Fe and Zn concentrations (ii). For the 0 Cu conditions, no Cu was added to the media and the Cu chelator, Bathocuproine (50 µM) was present. Total, shoot and root fresh weight is shown; the means (+/- SEM) were based on six plates, with four seedlings per line, per plate, each plate containing four plant lines. Means not sharing a letter are significantly different ($P \leq 0.05$); Tukey post-hoc test.

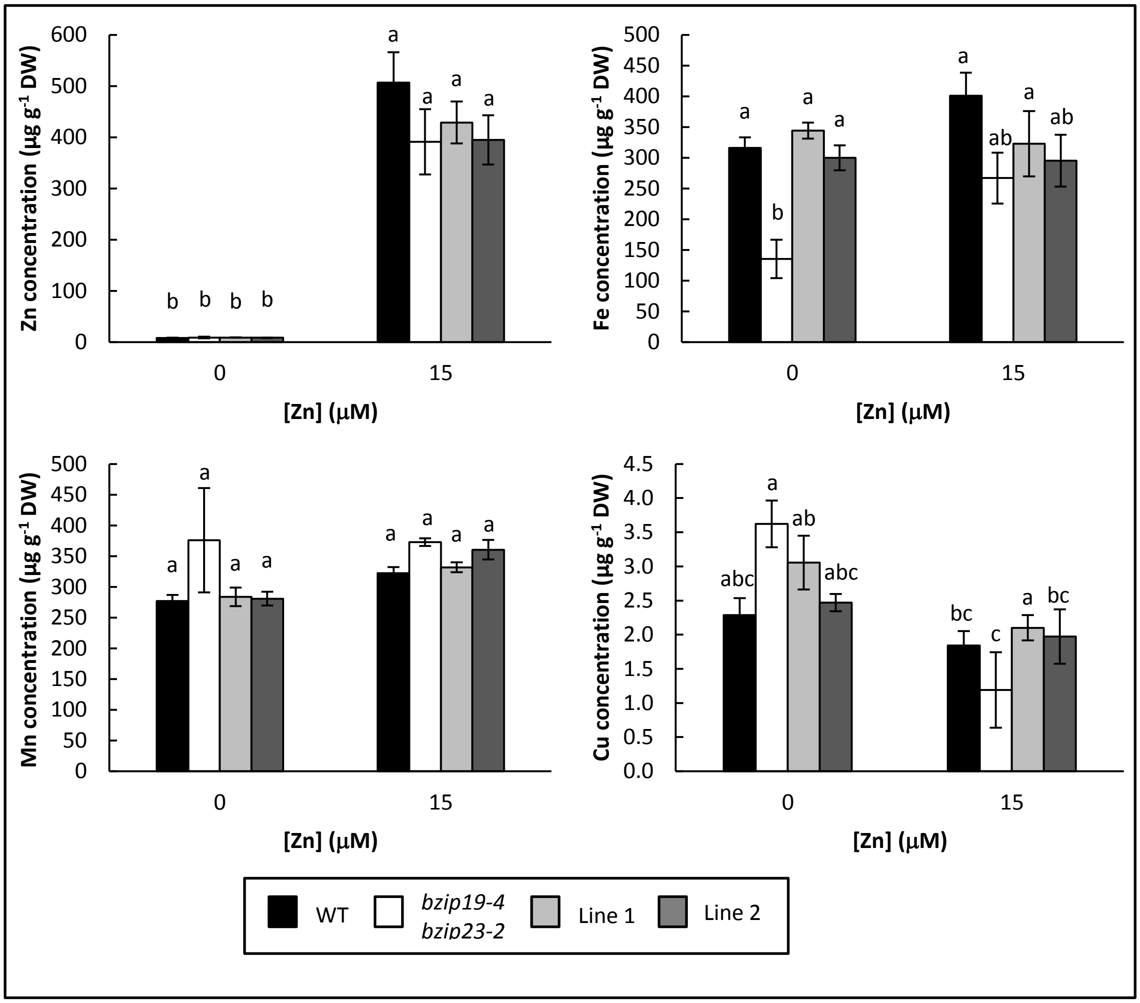

Fig. S7 Metal concentrations in wild type (WT), *bzip19-4 bzip23-2* double mutant, and bZIP56-expressing double mutant lines. Plants were grown on half MS media for 21 days with 0 µM or 15 µM Zn. Data are means (+/- SEM) of four biological replicates. Means not sharing a letter are significantly different ($P \leq 0.05$); Tukey post-hoc test.

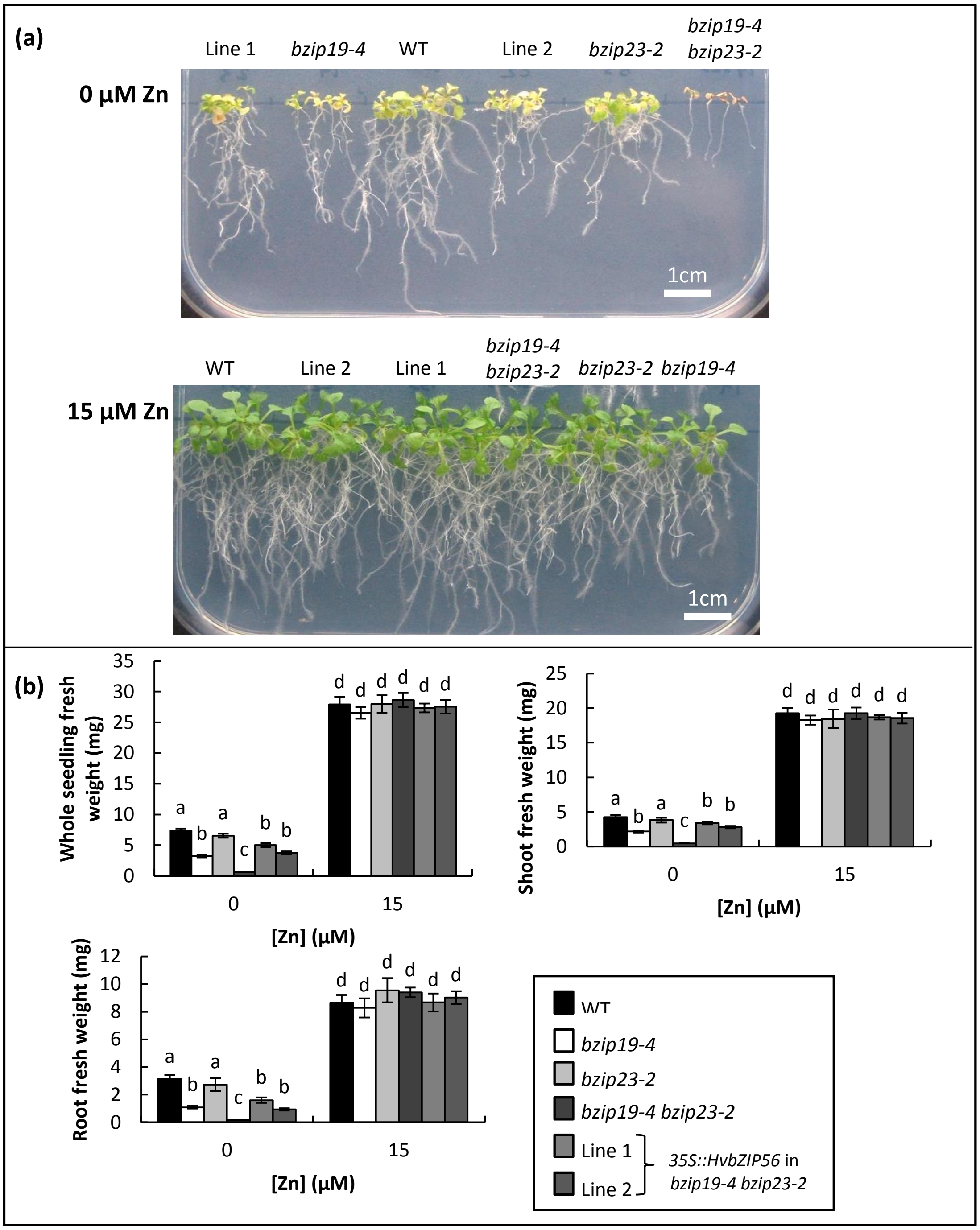

Fig. S8 HvbZIP56-expressing *bzip19-4 bzip23-2* lines grow to a similar level to *bzip19-4* single mutant under Zn-deficiency. Wild type (WT), *bzip19-4 bzip23*-2 mutants and transgenic lines grown on half MS media for 21 days with 0 µM Zn or 15 µM Zn. (a) Images of representative plates; (b) total, shoot and root fresh weight are shown for each construct. The means (+/- SEM) were based on six plates, with four seedlings per line, per plate, each plate containing four plant lines. Means not sharing a letter are significantly different (*P*≤0.05); Tukey post-hoc test.

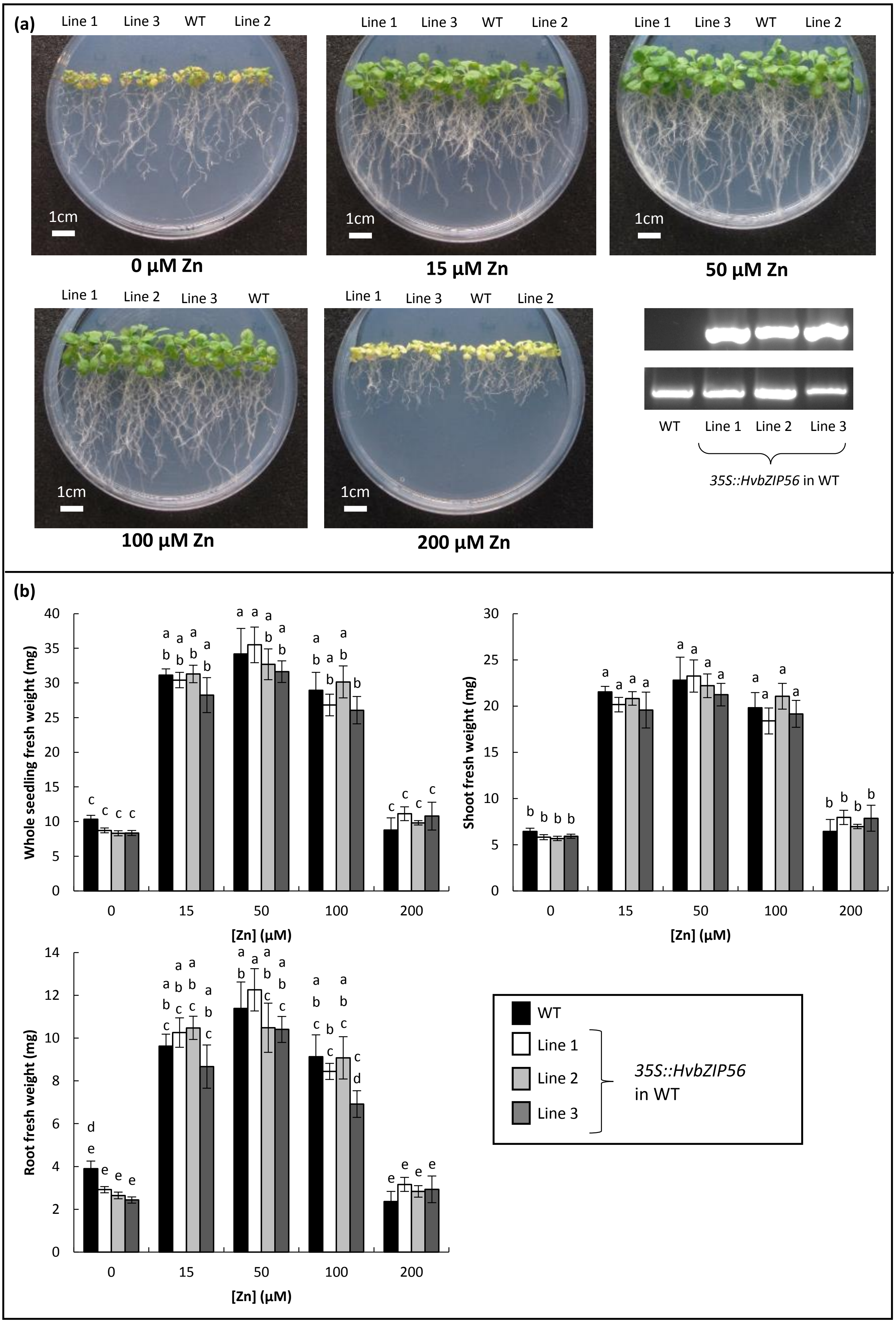
(a)
Line 1 Line 3 WT Line 2
1cm
0 µM Zn
Line 1 Line 3 WT Line 2
1cm
15 µM Zn
Line 1 Line 3 WT Line 2
1cm
50 µM Zn
Line 1 Line 2 Line 3 WT
1cm
100 µM Zn
Line 1 Line 3 WT Line 2
1cm
200 µM Zn
WT Line 1 Line 2 Line 3
35S::HvbZIP56 in WT
(b)
Whole seedling fresh weight (mg)
0 5 10 15 20 25 30 35 40
0 15 50 100 200
[Zn] (µM)
Shoot fresh weight (mg)
0 5 10 15 20 25 30
0 15 50 100 200
[Zn] (µM)
Root fresh weight (mg)
0 2 4 6 8 10 12 14
0 15 50 100 200
[Zn] (µM)
WT
Line 1
Line 2
Line 3
35S::HvbZIP56 in WT

Fig. S9 HvbZIP56 expression in wild type Arabidopsis does not influence their response to Zn deficiency or Zn excess. a) Images of representative plates and expression of HvbZIP56 in wild type (WT) with upper panel showing products for *HvbZIP56* and lower panel shows products for actin. (b) Wild type (WT) and *35S::HvbZIP56*-expressing lines grown on half MS media under a range of Zn concentrations. Total fresh weight is shown; the means (+/- SEM) were based on six plates with four seedlings per line, per plate, each plate containing three plant lines. Means not sharing a letter are significantly different ($P \leq 0.05$); Tukey post-hoc test.

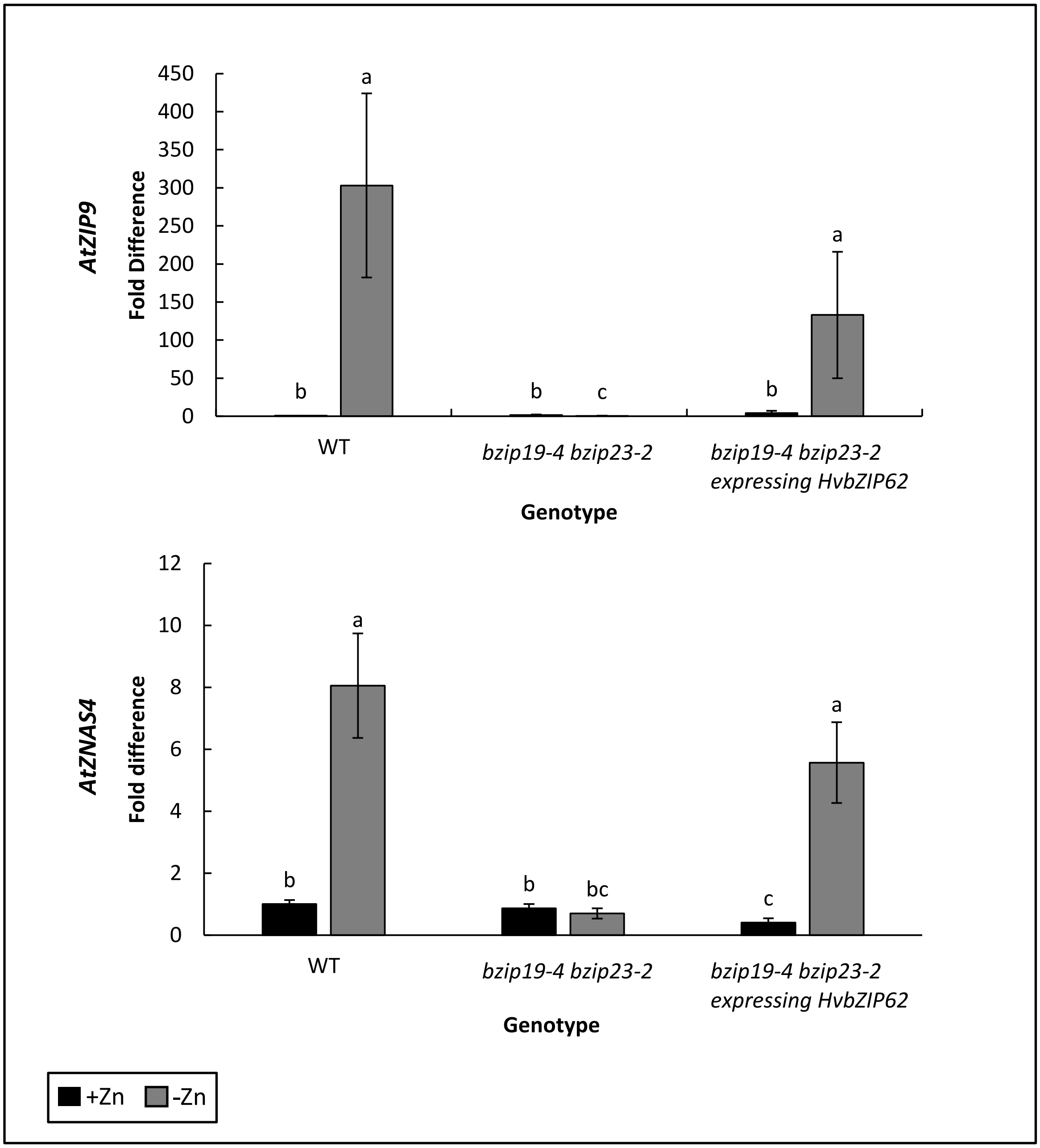

Figure S10. HvbZIP62 restores gene expression in response to Zn-deficiency in the Arabidopsis *bzip19-4 bzip23-2* mutant. Real-time PCR to compare Arabidopsis gene expression in response to Zn deficiency in wild type (WT), *bzip19-4 bzip23-2* double mutant*,* and *HvbZIP62*-expressing *bzip19 bzip23* line 1. Seedlings were grown for 3 weeks on MS medium with Zn (black) or without Zn (grey). *SAND* was used for normalization. The expression levels were relative to that in wild-type (WT) seedlings grown with Zn, which was expressed as 1. Data presented are means (+/- SEM) of three biological replicates. Means not sharing a letter are significantly different ($P \leq 0.05$); Fisher's Least Significant Difference (LSD) post-hoc test.

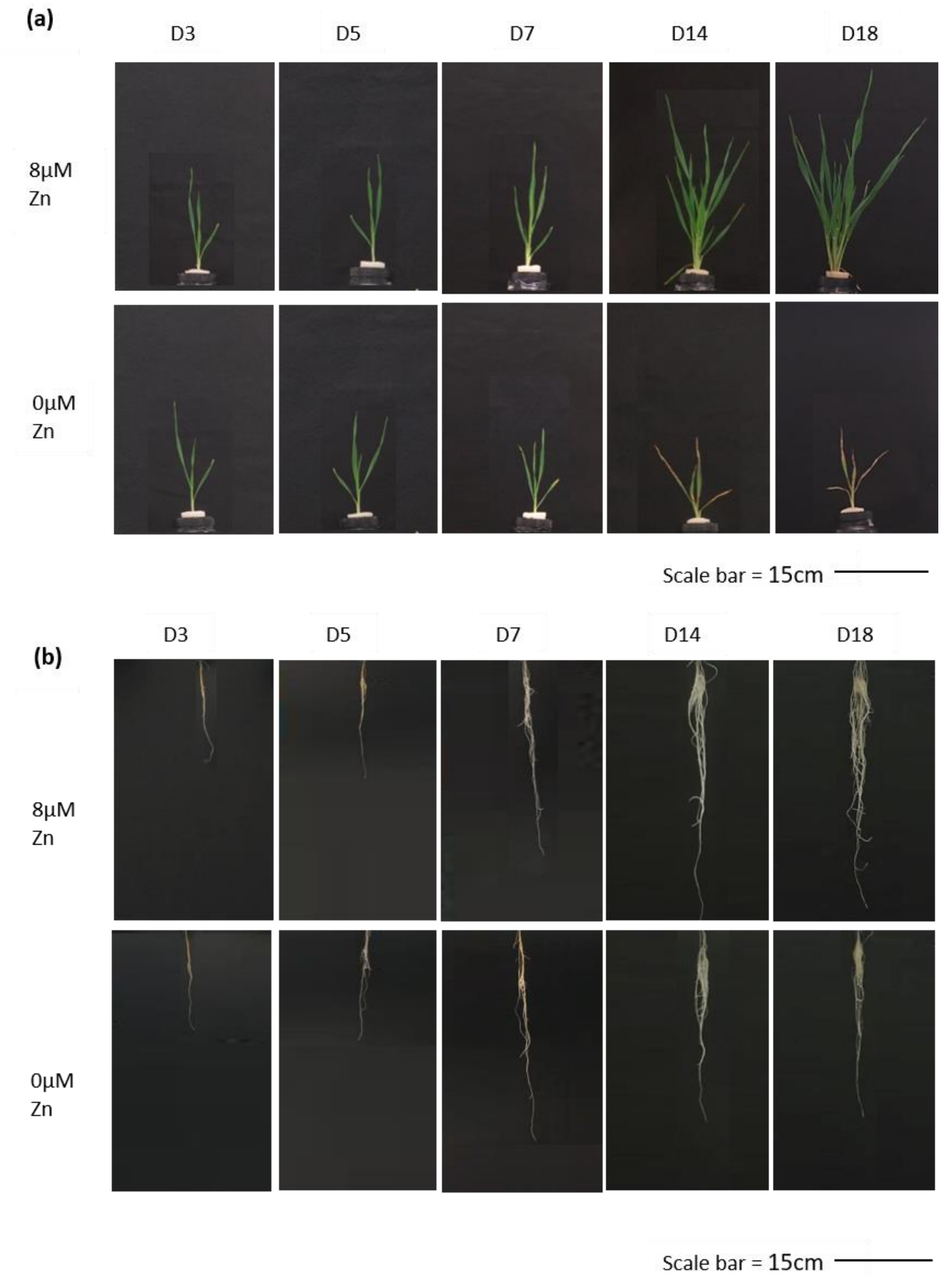
(a)
D3
D5
D7
D14
D18
8µM Zn
0µM Zn
Scale bar = 15cm
(b)
D3
D5
D7
D14
D18
8µM Zn
0µM Zn
Scale bar = 15cm

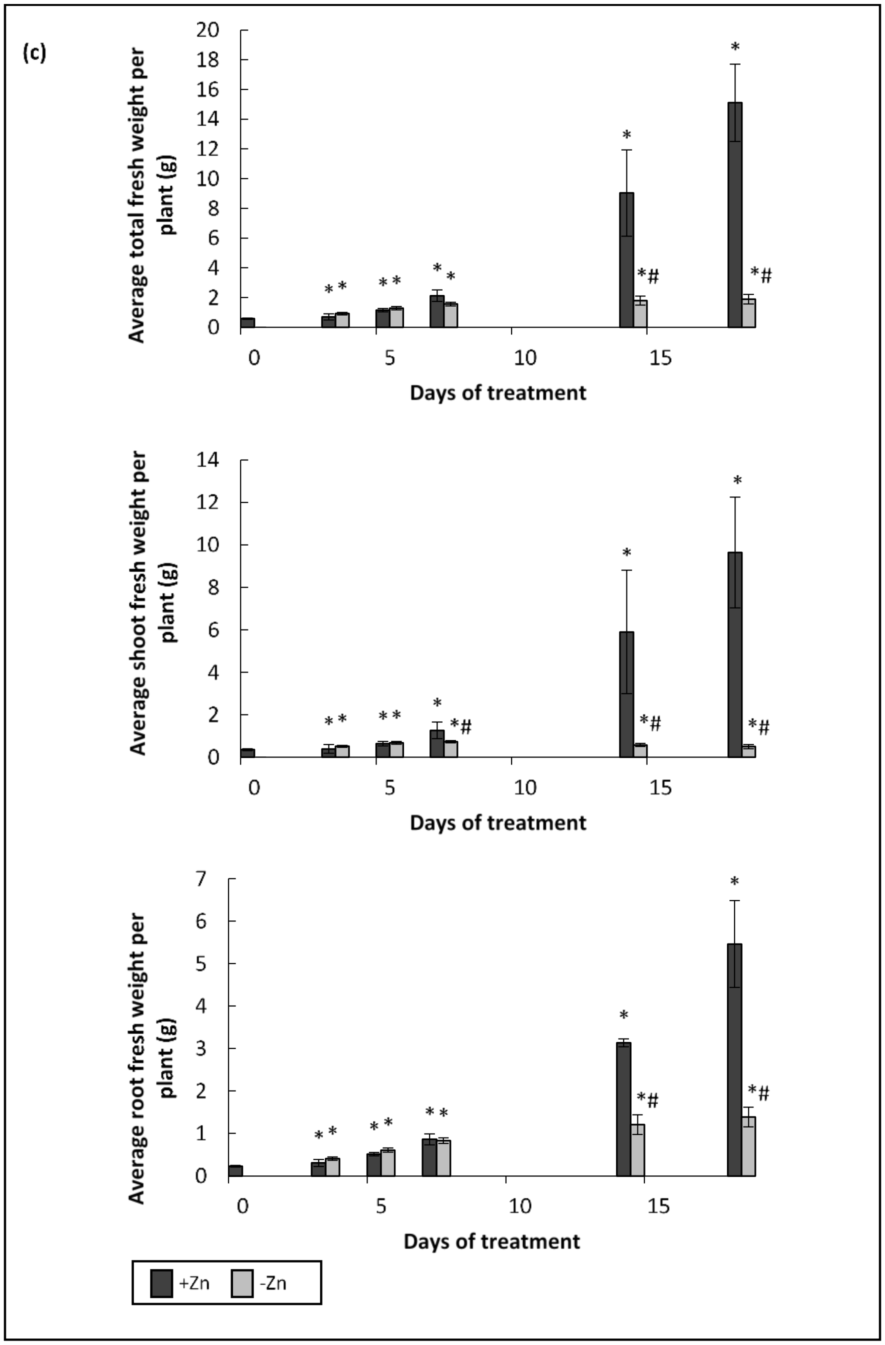

Fig. S11 Barley displays deficiency symptoms when grown under Zn-deficient conditions. Barley was grown on normal Zn (8 µM Zn) for 7 days before they were treated with either 8 µM Zn (+Zn) or 0 µM Zn (-Zn) for 0, 7 and 14 days. Photographs of shoots (a) and roots (b) and mean fresh weight measurements per plant (+/- SEM) for total fresh weight, shoot weight and root weight (c). #, $P \leq 0.05$ = significantly different to the mean of Zn-treated plants (+/- SEM). *, $P \leq 0.05$ = significantly different to the mean of Day 0 plants (+/- SEM); Tukey post-hoc test.

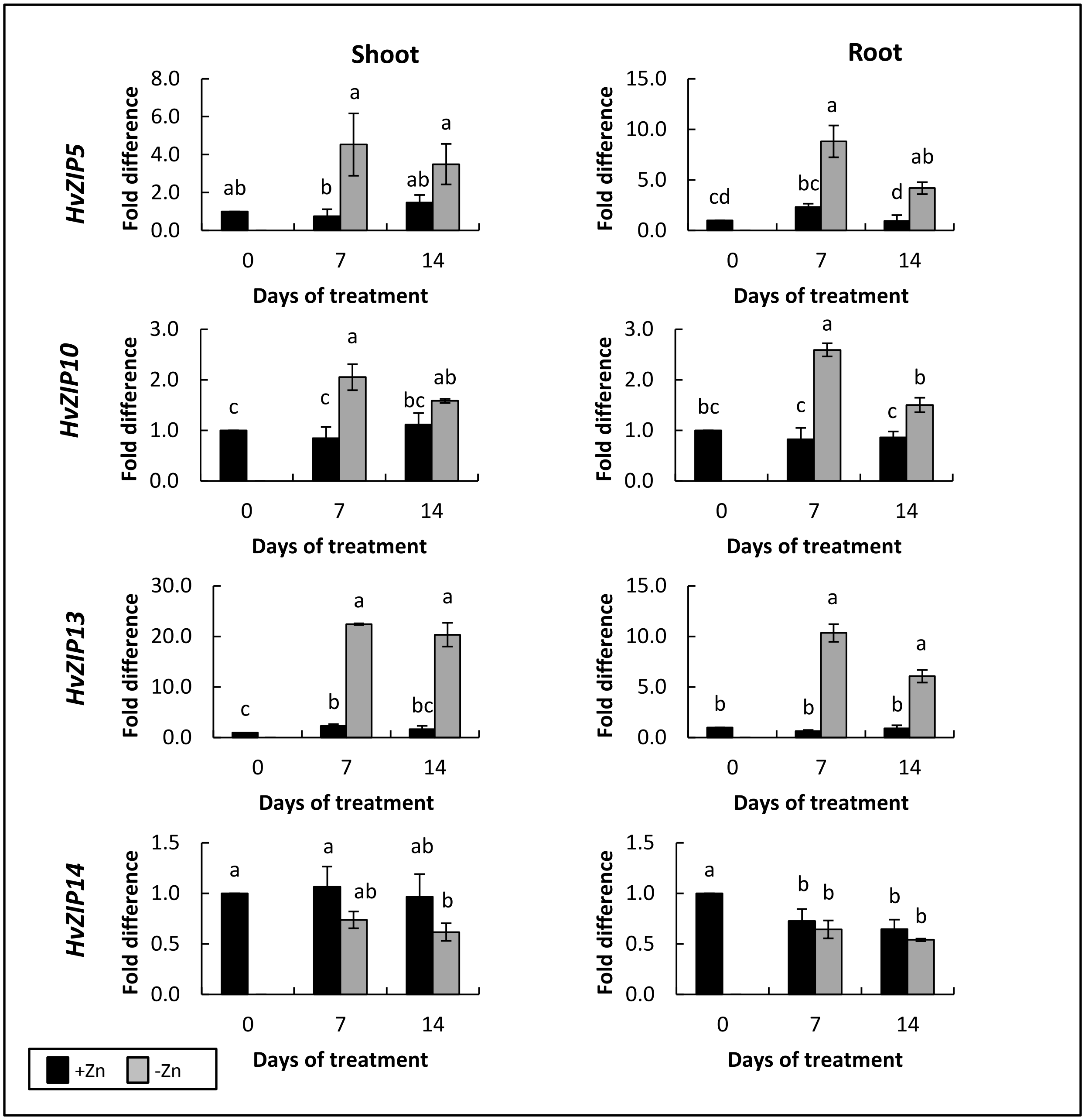

Fig. S12 Regulation of ZIPs following imposition of Zn-deficiency conditions. Real-time PCR determining gene expression of barley ZIPS in roots and shoots over time in response to Zn deficiency. The gene relative expression levels were calculated based Pfaffl (2001), standardised by normalizing to *HvRNABP* (Mikkelsen et al. 2012) and analyzed using Opticon software. Expression levels were relative to expression at day 0 of the treatment which was expressed as 1. All data are means (+/- SEM) of three biological replicates. Black bar and grey bar indicate gene expression level under 8 µM Zn (+Zn) and 0 µM Zn (-Zn) conditions respectively. Means not sharing a letter are significantly different ($P \leq 0.05$); Fisher's Least Significant Difference (LSD) post-hoc test.

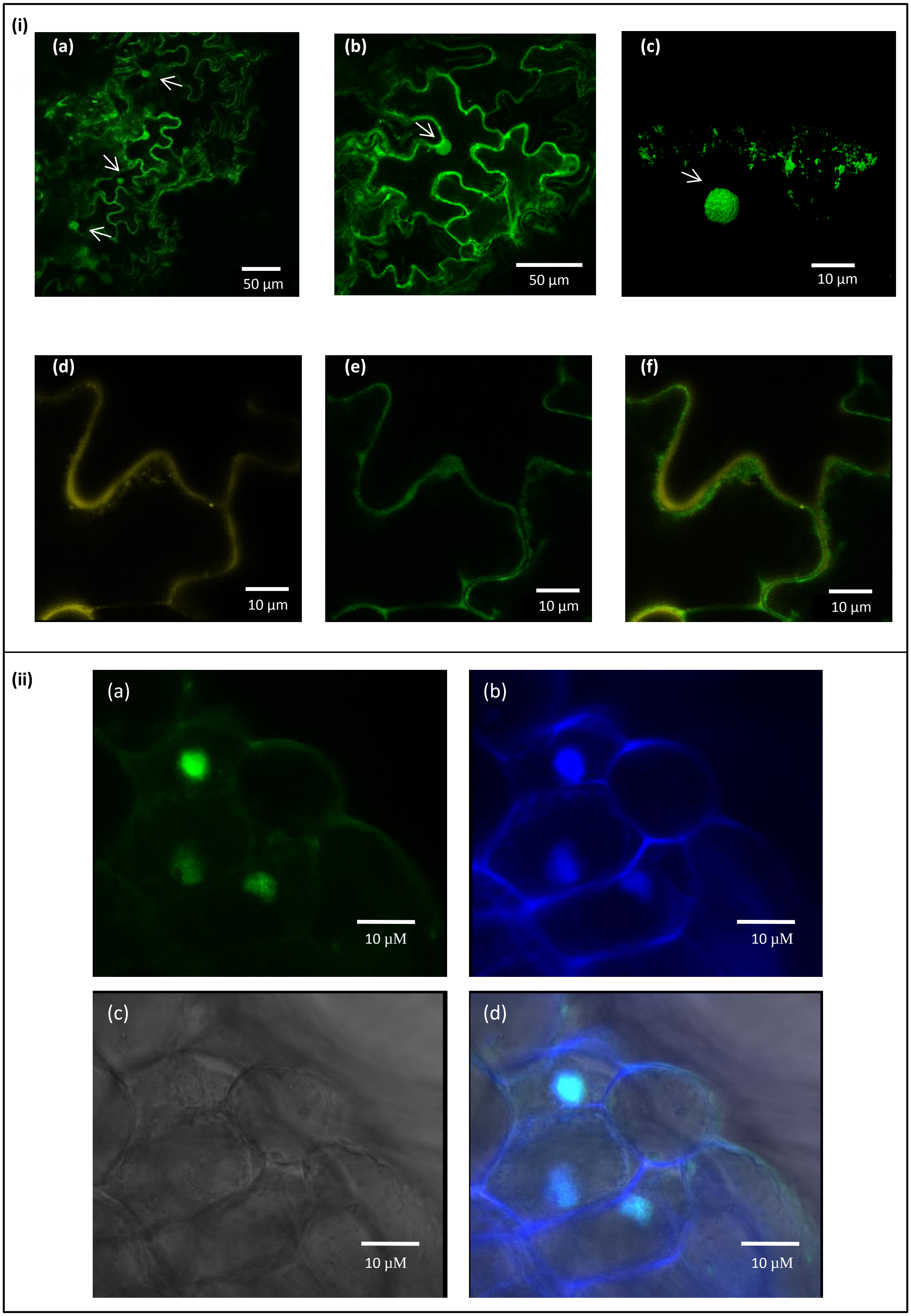

(i)
(a)
50 µm
(b)
50 µm
(c)
10 µm
(d)
10 µm
(e)
10 µm
(f)
10 µm
(ii)
(a)
10 µM
(b)
10 µM
(c)
10 µM
(d)
10 µM

Fig. S13 Localisation of HvbZIP56-GFP in tobacco (i). Confocal images: Top panel: Transient expression of *35S::HvbZIP56-GFP* in tobacco showing localisation in the cytoplasm and nucleus at increasing levels of magnification (a,b,c). Lower panel: localisation of *35S::HvbZIP56-GFP* (green) in cytoplasm (d) and *35S::*LTI6b-mOrange2 (orange) at the plasma membrane (e), the overlay showing LTI6b-mOrange2 is external to HvbZIP56-GFP (f). (ii) Colocalisation of HvbZIP56-GFP (a; shown in green) with a nucleus of root cell stained with Hoechst 33342 (b; shown in blue). The images were taken from the 10-day old Arabidopsis *bzip19-4 bzip23-2* mutant. The DIC image (c) and the overlay (d) of the same root cell were also taken.

Table S1 Primer purpose, nomenclature and sequence

**Purpose: Genotyping *bzip19* and *bzip23* Arabidopsis mutants.**		
**No**	**Primer name**	**Sequence (5' to 3')**
1	LBa1	TGGTTCACGTAGTGGGCCATCG
2	LBb1	GCGTGGACCGCTTGCTGCAACT
3	bZIP19F2	CTGTTTAGTGCGCCTTTAT
4	bZIP23R2	AAGGAACCTGAGCGAAGCTG
5	bZIP23F2	TCTTAAACCCTTCTCGCCGT
6	bZIP23R2	CAAACTGCTTTCGCTGCTCG
7	Actin2.F	GGTAACATTGTGCTCAGTGGTGG
8	Actin2.R	CTCGGCCTTGGAGATCCACATC
**Purpose: Barley *bZIP* genes GATEWAY cloning**		
**No**	**Primer name**	**Sequence (5' to 3')**
9	HvbZIP1Topo_F	CACCATGGACGACGGGCACCTC
10	HvbZIP1Topo_S	CTAATGAAAACACGTATGAGG
11	HvbZIP10Topo_F	CACCATGGACGACAACGGGGAC
12	HvbZIP10Topo_S	TCACCTCTTTACATCATCTGGCA
13	HvbZIP55Topo_F	CACCATGGACGACGGACTATAC
14	HvbZIP55Topo_S	TTAGGCAACAGAGTTCACGAAGC
15	HvbZIP56Topo_F	CACCATGGACGACGGGGACATC
16	HvbZIP56Topo_S	TCATTATAGCATCCTAGCTAACG
17	HvbZIP56Topo_NS	CAGCATTTGGCCCCCACGG
18	HvbZIP57Topo_F	CACCATGGACGACGGGGTGGAC
19	HvbZIP57Topo_S	TCAGCTAGGGAAGCAGAGCTC
20	HvbZIP58Topo_F	CACCATGGACGACGGGGACCTG
21	HvbZIP58Topo_S	CTAACGAAAACAGACAGGAGG
22	HvbZIP62Topo_F	CACCATGGATGACGGGGACCTC
23	HvbZIP62Topo_S	TCACTGCTTTTCGGAACTGGG
**Function: Real time PCR, barley *bZIP* and *ZIP* expression analysis**		
**No**	**Primer name**	**Sequence (5' to 3')**
24	HvRNABP F	CGCCCAGTTATCCATCCATCTA
25	HvRNABP R	AAAAACACCACAGGACCGGAC
26	HvGADPH F	GCTCAAGGGTATCATGGGTTACG

27	HvGADPH R	GCAATTCCAGCCTTAGCATCAAAG
28	HvZIP5rtF	ATCATCGGCATGTCCTTGGG
29	HvZIP5rtR	AAGAAAGACTTGTGGCGAAACC
30	HvZIP13rtF	GCTCGGCATCAACATCTCC
31	HvZIP13rtR	GTTGTAGGCTTGCGGCTAG
32	HvZIP14rtF	CACAGATGCACGATCAGAGAAC
33	HvZIP14rtR	CCACAATATCCACGGAACTCATA
34	HvbZIP1rtF	ACGACATCCTCATGGACACG
35	HvbZIP1rtR	TAATCTGGGCACCGCCCT
36	HvZIP10rtF	GACCTCATTGCTGCTGATTT
37	HvZIP10rtR	AGCTAGGCAACAGGTCGTAGT
38	HvbZIP55rtF	TCTGTCGTCTGTACTGCTGC
39	HvbZIP55rtR	ACAGCATCCACCACTTCAGG
40	HvbZIP56rtF	TGCCGGTGACACTATGGGTT
41	HvbZIP56rtR	TTACAGCATTTGGCCCCCA
42	HvbZIP57rtF	ATTGCGGGCTCGATGAAGAT
43	HvbZIP57rtR	GCTAGGGAAGCAGAGCTCAA
44	HvbZIP58rtF	GTCAGGGGCAGGATCGAAG
45	HvbZIP58rtR	TGAGCTCATCATGACCGGG
46	HvbZIP62rtF	CATTGGGGGTGCGAAATCTG
47	HvbZIP62rtR	GAACTGGGTAAACAGCCGGA
**Function: Real time PCR, Arabidopsis *ZIP* expression analysis**		
48	AtSANDrtF	AACTCTATGCAGCATTTGATCCACT
49	AtSANDrtR	TGATTGCATATCTTTATCGCCATC
50	AtZIP2rtF	TAATAACAACCACGTCGGAG
51	AtZIP2rtR	AGCAAAGCTGTGTCTCCAAA
52	AtZIP4rtF	CACGGACATATGCACGGGAA
53	AtZIP4rtR	GACACAATCCCGAGCTCCAA
54	AtNAS4rtF	TCGGATCTCGCGTGTAACTG
55	AtNAS4rtR	CACCTGCGAACTCCTCGATA
56	AtDEFL203rtF	TAATGGACTCCCAAAGGCGG
57	AtDEFL203rtR	ACTTCACATGCCCCAAACCA
58	AtZIP9rtF	CAATAATCATAGGAATATCGCTTGG
59	AtZIP9rtR	AGAAAGCCATCATGGCAGAT
60	AtZIP12rtF	CAATGTTGATTGAATCCTTTGC
61	AtZIP12rtR	CCATGAGAATGTCCTTGTGA

Table S2 HvbZIPs identified from bioinformatics analysis

	Contigs	Position	NCBI cDNA accession No. (cultivar)	MIPs[1] cDNA accession No. (cultivar)	Amplification/Cloning	Molecular weight (Da) of GP sequence
HvbZIP1	- morex_contig_11993 (Full-length)<br>- bowman_contig_63503 (Full-length and identical to the Morex background)<br>- barke_contig_276336 (partial sequence)	Chromosome 7HL :33,218,969 - 33,219,416	Not found	MLOC_2245.1 (Morex)	Yes/Yes	19,857
HvbZIP10	- morex_contig_1563657 (Full-length)<br>- bowman_contig_846659 (Full-length and identical to the Morex background)<br>- barke_contig_268371 ((Full-length but 3 bp longer than found in Morex or Bowman)	Chromosome 7H: 536,391,693-536,392,374	Not found	MLOC_12585.1 (Morex)	Yes/Yes	24,329
HvbZIP55	- morex_contig_136513 (Full-length)<br>- bowman_contig_63503 (Full-length and identical to the Morex background)<br>- barke_contig_282304 and 486135 (partial sequences)	Chromosome 3H: 461,941,056-461,941,698	AK354846.1 (Haruna Nijo)	MLOC_5655 (Morex)	Yes/Yes	23,011
HvbZIP56	- morex_contig_38563 (Full-length)<br>- bowman_contig_1981097 (Full-length and identical to the Morex background)<br>- barke_contig_1785171 (partial sequence)	Chromosome 7H: 591,350,097-591,350,849	AK253086.1 (Haruna Nijo)	MLOC_53694.1 (Morex)	Yes/Yes	27,243
HvbZIP57	- morex_contig_1565570 (Full-length)<br>- bowman_contig_1995766 (Full-length and identical to the Morex background)	Chromosome 1H: 35,678,456-35,679,076	AK371968.1 (Haruna Nijo)	MLOC_13410 (Morex)	Yes/Yes	23,972
HvbZIP58	- morex_contig_236589 and 1591639 (partial sequences)<br>- bowman_contig_1978011 and 123086 (partial sequences)	Chromosome7HL: 38,634,376-38,634,982	AK354735.1 (Haruna Nijo)	Not found	Yes/Yes	21,819
HvbZIP61	Not found	Not found	AK361769.1 (Haruna Nijo)	Not found	No/No	35,779*
HvbZIP62	- morex_contig_44880 (Full length)<br>- bowman_contig_849113 (Full-length but 9 bp longer than found in Morex)	Chromosome 5H: 9,201,389-9,202,168	Not found	MLOC_60894.1 (Morex)	Yes/Yes	27,539

* molecular weight predicted from the Haruna Nijo sequence.

Table S3. Accession numbers for sequences used in the phylogenetic analysis in Figure 1.

Species	Nomenclature	Accession No.
Wheat	TabZIP4	Traes_1AL_00A8A2030.2
	TabZIP33	Traes_5AS_6F02C9967.1
	TabZIP55	Traes_7AL_A8CAE984E.1
	TabZIP56	Traes_7AL_C7CF7087B.2
	TabZIP62	Traes_1BL_1A885E733.1
	TabZIP79	Traes_3B_34C2CD876.2
	TabZIP97	Traes_5BS_FC54F9BEA.1
	TabZIP120	Traes_7BL_096916DC5.1
	TabZIP150	Traes_5DS_4F7973584.1
	TabZIP179	Traes_7DL_70D4FDB2A.1
	TabZIP180	Traes_7DL_7DDA16622.3
Rice	OsbZIP7	LOC_Os01g58760
	OsbZIP44	LOC_Os05g41540
	OsbZIP48/53	LOC_Os06g50310
Arabidopsis	AtbZIP19	At4G35040
	AtbZIP23	At2G16770.1
	AtbZIP24	At3G51960
Brachypodium	BdbZIP11	Bradi1g30140.1
	BdbZIP32	Bradi2g21197
	BdbZIP33	Bradi2g21200
	BdbZIP44	Bradi2g52590
Cucumber	CsbZIP59	XP_004147043
	CsbZIP60	XP_004144846
Barley	HvbZIP1	MLOC_2245.1
	HvbZIP10	MLOC_12585.1
	HvbZIP55	MLOC_5655
	HvbZIP56	MLOC_53694.1
	HvbZIP57	MLOC_13410
	HvbZIP58	BAJ85954.1
	HvbZIP61	BAJ92973.1
	HvbZIP62	MLOC_60894.1

Wheat F group sequences (Li et al. 2015); Rice and Brachypodium (Liu and Chu 2015); Arabidopsis (Assuncao et al. 2010); Cucumber (Baloglu et al. 2015). The barley sequences used in the phylogenetic tree are mainly those cloned from Golden Promise in this study; the accession numbers given above are for sequences from Morex and Haruna Nijo.

Table S4 Percentage identity/similarity of Arabidopsis, and barley F group bZIP

Sequence	AtbZIP19	AtbZIP23	AtbZIP24	HvbZIP1	HvbZIP10	HvbZIP55	HvbZIP56	HvbZIP57	HvbZIP58	HvbZIP61/ BA92973	HvbZIP62
**AtbZIP19**		69.5	28.4	33.0	34.9	33.6	43.3	38.6	34.4	8.3	41.4
**AtbZIP23**	80.3		32.6	33.0	38.3	35.6	41.5	39.9	36.6	9.4	44.7
**AtbZIP24**	45.2	44.1		28.2	25.1	27.5	30.7	28.2	33.8	10.8	29.4
**HvbZIP1**	44.3	43.3	43.1		42.9	33.1	45.8	33.7	72.9	9.7	42.4
**HvbZIP10**	48.1	52.9	36.9	49.3		31.4	56.4	28.7	36.3	9.3	50.5
**HvbZIP55**	46.6	47.0	41.6	41.8	41.7		36.5	50.2	36.2	10.1	30.6
**HvbZIP56**	58.5	52.9	45.1	52.7	65.6	53.6		39.9	45.1	7.5	65.1
**HvbZIP57**	47.4	49.6	41.8	43.4	39.8	58.0	48.2		36.1	8.8	39.3
**HvbZIP58**	44.0	49.1	47.1	79.3	43.6	46.9	51.9	41.7		9.5	42.5
HvbZIP61/ BA92973	16.2	15.4	18.5	15.1	13.8	15.4	13.8	15.9	13.3		9.7
**HvbZIP62**	55.9	59.5	43.2	48.8	57.8	39.7	71.7	49.6	49.3	13.3	

Top diagonal none-shaded = percent identity. Bottom diagonal = percent similarity. HvbZIP61/BA92973 is included for comparison but may not be a barley gene (see text).

Table S5 Nomenclature of mutant lines

**Line Number**	**SALK Number**	**Segregation Status supplied**	**Gene**	**Assuncao et al. (2010) mutant name**	**Inaba et al. (2015) mutant name**	**Proposed mutant name by this study**
N667534[1]	SALK_144252C	Homozygous	*bZIP19*	*bzip19-1*	*N/A*	*bzip19-1*
N657869	SALK_005336C	Homozygous	*bZIP19*	N/A	*bzip19-1*	*bzip19-4*
N506692	SALK_006692	Segregating	*bZIP19*	N/A	*bzip19-2*	*bzip19-2*
N583399	SALK_083399	Segregating	*bZIP19*	N/A	*bzip19-3*	N/A
N656437	SALK_045200C	Homozygous	*bZIP23*	*bzip23-1*	*bzip23-1, bzip23-2 (in different parts of paper)*	*bzip23-1*
N653060	SALK_018248C	Homozygous	*bZIP23*	N/A	*bzip23-1, bzip23-2 (in different parts of paper)*	*bzip23-2*

[1] Homozygous line replacing the N644252 retired line

Table 6 ZDRE motifs and position

Barley ZIP/bZIP	No. of potential ZDRE motifs	Closest sequence to the ZDRE (5' to 3') RTGTCGACAY (cultivar)	Position (upstream from the start codon) ZDRE (closest sequence)
HvIRT1	1	CTGTCGCCAC (morex/bowman)	1186 bp
HvZIP1	1	ATGTCTCGAG (morex/bowman	295 bp
HvZIP2	1	CTGACGACAC (morex/bowman	730 bp
HvZIP3	2	GTGTCAACAC (morex/bowman)<br>GTGTCGACAA* (morex/bowman)	546 bp<br>1604 bp
HvZIP5	2	GTGTCGACAC* (morex/bowman)<br>GTGTCGACAC* (morex/bowman)	689 bp<br>928 bp
HvZIP6	2	GTTTCGACAC (morex/bowman)<br>ATGTGCAAAG (morex/bowman)	186 bp<br>311 bp
HvZIP7	1	ATGTCGACAT* (morex/bowman)	672 bp
HvZIP8	1	GTGTCGACAC* (morex/bowman)	658 bp
HvZIP10	1	GTGTCGACAC* (morex/bowman)	443 bp
HvZIP11	1	ATGTCGGCTT (morex/bowman)	165 bp
HvZIP13	1	ATGTCGTCAC (morex/bowman)	243 bp
HvZIP14	2	AAGTCAACAA (morex/bowman)<br>ATGTCTTGAC (morex/bowman)	187 bp<br>1055 bp
HvZIP16	1	ATGCCCACAC (morex/bowman)	496 bp
HvbZIP1	1	ATGTCGACAT* (morex/bowman)	113 bp
HvbZIP10	1	ATGACGACAT (morex/bowman)	397 bp
HvbZIP55	1	TGTCTAGACA (morex/bowman)	1022 bp
HvbZIP56	1	ATGTCATCGA (morex/bowman)	988 bp
HvbZIP57	1	TGTCGTCCGA (morex/bowman)	131 bp
HvbZIP58	1	ATGTCGACAC*(morex/bowman)	106 bp
HvbZIP61	N/A	N/A	No promoter sequence was found
HvbZIP62	1	ATGCCCACAT (morex/bowman)	749 bp

* = ZDRE motif palindromic
